# TorpeDNA: a fit-for-purpose eDNA sampling device for marine biodiversity monitoring across applications and scales

**DOI:** 10.7717/peerj.21390

**Published:** 2026-06-22

**Authors:** Xavier Pochon, Teddy Urvois, Erwan Quéméré, Yann Reynaud, Erin Bomati, Olivier Laroche, Sophie Arnaud-Haond, Verena M. Trenkel, Loïc Baulier, Germain Boussarie, Pablo Saenz-Agudelo

**Affiliations:** 1 Molecular Surveillance Team, Biosecurity Group, Cawthron Institute, Nelson, New Zealand; 2 Citizens of the Sea, Nelson, New Zealand; 3 Institute of Marine Science, University of Auckland, Auckland, New Zealand; 4 DECOD (Ecosystem Dynamics and Sustainability), Institut Agro, INRAE, IFREMER, Rennes, France; 5 Microbiology Health and Environment Laboratory, LSEM, IFREMER, Nantes, France; 6 MARBEC, Université de Montpellier, CNRS, Ifremer, IRD, Université de Montpellier, Sète, France; 7 DECOD (Ecosystem Dynamics and Sustainability), Institut Agro, INRAE, IFREMER, Nantes, France; 8 DECOD (Ecosystem Dynamics and Sustainability), Institut Agro, INRAE, IFREMER, Lorient, France

**Keywords:** Citizen science, Environmental DNA (eDNA), Marine biodiversity monitoring, Metabarcoding, Open-ocean observation, Plankton communities, Scalable monitoring tools, Towed sampling device, TorpeDNA, Bacterial ecology

## Abstract

**Background:**

Environmental DNA (eDNA) has revolutionized biodiversity monitoring by enabling species detection from microbes to megafauna without direct observation or capture. Yet most marine eDNA collection systems remain expensive and labor-intensive, limiting their deployment at scale or by non-specialists. To address these challenges, we developed TorpeDNA, a compact, low-cost, and user-friendly eDNA sampler designed to operate under diverse marine conditions, including high-speed towing and citizen-science use. This proof-of-concept study evaluates its performance, scalability, and limitations across three contrasting marine environments encompassing different trophic levels, taxa, and user contexts.

**Methods:**

Three case studies were conducted. (1) In Northern Brittany (France), TorpeDNA (20 µm filters) was compared with large-capacity filtration capsule (0.45 µm) for characterising eukaryotic and vertebrates’ communities using metabarcoding. (2) Along the French Atlantic coast, TorpeDNA was benchmarked against serial laboratory filtration (10–0.45 µm fraction) to assess bacterial assemblages near aquaculture and wastewater gradients. (3) In the South-West Pacific, TorpeDNA was deployed aboard a *Citizens of the Sea* vessel to sample open-ocean plankton communities from microbes to vertebrates along a 2,000 km transect between Aotearoa-New Zealand and Fiji.

**Results:**

Across all studies, TorpeDNA generated biodiversity profiles broadly comparable to those from fine-pore or serial filtration methods. However, some notable differences were observed between methods, largely attributable to the different filter pore sizes and water volumes processed, which influenced the preferential capture or exclusion of host-associated or sediment-bound microbes, larger plankton forms, and/or rare taxa. TorpeDNA effectively recovered dominant microbial, phytoplankton, and zooplankton assemblages, reproducing known ecological and biogeographic gradients, from temperate to tropical plankton turnover and coastal-to-offshore transitions. Vertebrate DNA detection was limited in both methods, likely reflecting low natural eDNA concentrations, insufficient water volume processed, or marker inefficiency and highlighting the need for further optimization of sampling duration, flow rate, and assay sensitivity.

**Conclusion:**

TorpeDNA achieves a practical balance between usability, versatility across trophic levels, and integration across spatial and methodological scales. Its robust, low-cost design and compatibility with different filter types and standard molecular workflows make it scalable across platforms, from small to large research vessels to citizen-science initiatives and offering a fit-for-purpose tool for global, inclusive, and scalable ocean biodiversity sampling.

## Introduction

Environmental DNA (eDNA) has emerged as a transformative tool for biodiversity assessment across biomes, offering unprecedented insights into the complexity of ecosystems (Deiner et al., 2017; Ruppert, Kline & Rahman, 2019; Beng & Corlett, 2020). Environmental DNA refers to the genetic material originating from organisms and released into the environment *via* skin cells, mucus, gametes, faeces, decaying tissue, or as intact cells such as microbes, spores, or larvae. This DNA can exist in various states, from free-living (extracellular) DNA fragments to intact cellular material, or bound to organic particles and sedimentary matter (Pawlowski, Apothéloz-Perret-Gentil & Altermatt, 2020; Power et al., 2023). By analyzing genetic material shed by organisms into their environment, eDNA surveys enable the detection and monitoring of species without the need for direct observation or capture.

This approach has been applied successfully in various contexts, including biodiversity and conservation (Yang et al., 2021; Sahu et al., 2023), biosecurity and invasion biology (Zaiko et al., 2018; Jarman et al., 2024), and environmental health assessments (Wood et al., 2013; Cordier et al., 2021; Suren, Burdon & Wilkinson, 2024). The high-throughput molecular analysis of eDNA has proven particularly valuable in aquatic ecosystems, where traditional sampling methods often fall short in capturing the full scope of biodiversity (Wilkinson et al., 2024). Given the rapid changes in ecological stability driven by climate change and other anthropogenic stressors, eDNA-based approaches are becoming increasingly critical for informing conservation and management strategies (Aylagas et al., 2020; Cordier et al., 2021; Gold et al., 2022; Zhang, Zhao & Yao, 2023).

The heterogeneous nature of eDNA influences how it is transported, degraded, and ultimately bound in aquatic environments. A substantial fraction of eDNA binds to organic or sedimentary particles, making it effectively recoverable using filters with larger pore sizes (*e.g*., 5–20 μm) and thereby challenging the prevailing reliance on ultra-fine filtration (Turner et al., 2014; Sepulveda et al., 2019; Cooper et al., 2022; Zaiko et al., 2022; Pochon et al., 2024). Despite this, most traditional eDNA sampling protocols still depend on stationary or submersible pump systems equipped with fine-pore capsules to collect eDNA from the water column (Bowers et al., 2021; Peixoto et al., 2021; Patin & Goodwin, 2023; Jaquier et al., 2024). However, such systems are costly, complex, and time-consuming to deploy, with limitations that restrict their applicability at scale, especially in remote and dynamic marine environments. Instead, research studies could capitalize on the thousands of seafarers and vessel types traversing the world’s oceans each day to collect comprehensive biodiversity and time-series data (Lauro et al., 2014; de Vargas et al., 2022), but eDNA collection tools must be fit-for-purpose to be effectively used by non-specialist operators across diverse platforms. The development of portable and autonomous tools is needed to address these constraints by enabling continuous in-water eDNA collection directly onto enclosed filters during vessel towing.

To further expand eDNA sampling across vessel types and operating conditions, recent innovations have aimed to improve practicality, scalability, and cost-effectiveness. The Cruising Speed Net (CSN) exemplifies this effort, enabling surface sampling of eDNA material at sailing speeds of up to five knots (von Ammon et al., 2020, 2023; O’Rorke et al., 2022). However, its cost, bulk, and limited speed range all reduce its suitability for widespread deployment, particularly on small, fast-moving vessels. To address these constraints, we developed the TorpeDNA, a compact, low-cost, and semi-autonomous eDNA collection device designed for in-tow deployment at higher speeds. TorpeDNA captures eDNA directly onto enclosed filters during vessel movement using in-water eDNA collection technology (Pochon et al., 2024), making it ideal for scalable biodiversity surveys using recreational yachts, small crafts or research and commercial vessels.

The overall aim of this article is to introduce and provide the first multi-context evaluation of the TorpeDNA device across three distinct research settings and methodological comparisons. This compact, affordable system can operate at towing speeds of up to 12 knots, making it accessible to a wide range of users, from research institutions to citizen scientists, and adaptable to diverse aquatic environments. To assess its broader applicability, we conducted three case studies spanning coastal and open-ocean systems in France and across the South-West Pacific. Our research objectives were to (1) evaluate whether TorpeDNA recovers taxonomic diversity and community composition comparable to established eDNA sampling approaches across contrasting ecological and operational contexts, and (2) assess whether its design and operational simplicity support scalable deployment for distributed biodiversity monitoring, including citizen science initiatives. Together, these objectives allow us to evaluate the device’s performance, practical limitations, and potential contribution to scalable ocean biodiversity assessment.

## Materials and Methods

### TorpeDNA’s history, design & specifications

The TorpeDNA device was designed as a compact, lightweight, and cost-effective eDNA sampling tool to enhance accessibility and scalability in aquatic biodiversity monitoring. The device is deployed by towing it behind a moving vessel, fully submerged, using a tow line rather than being operated as a handheld sampler. The device was inspired by the 100 year-old *Plankton Indicator*, a small open torpedo with a removable filtering cod-end enabling fishermen to quickly determine areas with dense phytoplankton populations, hence increasing their chances of good fish catches (Hardy, 1926; Glover, 1953; Planque & Reid, 2002). The TorpeDNA was also designed to include the in-water eDNA capture capability previously developed for and tested with the CSN device (von Ammon et al., 2020; Pochon et al., 2024). An initial stainless-steel version of the TorpeDNA was built in 2022 as part of a collaboration between the Cawthron Institute and Sequench Ltd in Nelson, New Zealand. The latter metallic version, however, was over 4 kg in weight, and therefore was deemed too heavy for Citizen Science applications. Later, the Cawthron Institute contracted Kernohan Engineering Ltd (Nelson, NZ) for the design of a TorpeDNA prototype combining plastics and stainless-steel components, aimed at reducing both weight and towing drag. Initial field-testing indicated that this 2 kg-heavy version performed well at up to 12 knots speed, but it became apparent that some of its features created excess drag, making it hard to pull back the device upon recovery in the high-seas (Frankham & Robinson, 2024). Blender Ltd (Auckland, NZ) was then contracted to run computational fluid dynamics (CFD) analyses, which led to improved features and reduced the drag by 55% (Material S1).

**Figure 1:**
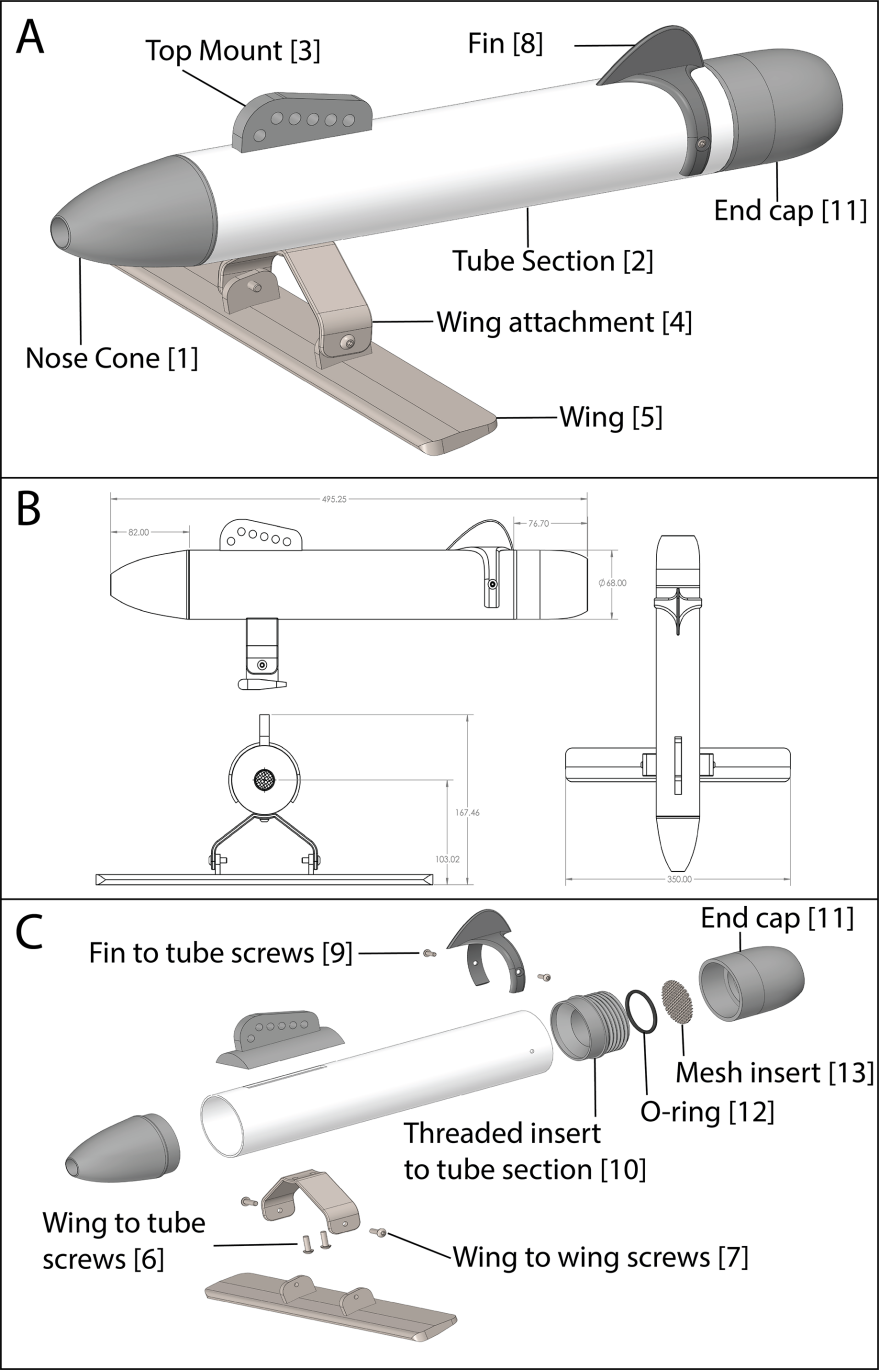
Design and specifications of the TorpeDNA device. (A) Assembled device ready for use, (B) parts measurements, and (C) exploded view. Numbers in square brackets correspond to the list of parts and material components detailed in Table S1.

The improved version of the TorpeDNA is lightweight (1.2 kg), has a streamlined torpedo-shaped body (Fig. 1A) and consist of 3D-printed components (nose, line attachment, back fin, and cod-end), a PVC tube (main body), and stainless-steel parts (wing and attachment). The device is approximately 50 cm long, 35 cm wide, 17 cm in height, and the main body is 68 mm in diameter (Fig. 1B). All 3D-printed components are permanently glued onto the PVC tube, except for the back fin, which is detachable (Fig. 1C). Also detachable *via* stainless-steel screws are the wing and attachment. The cod-end, consisting of a threaded back-end portion that includes a detachable stainless grid onto which a sterile eDNA filter (20 μm nylon, 47 mm; Merck Millipore Ltd., Darmstadt, Germany), can be added and locked in place with an O-ring (RS Pro, Auckland, New Zealand) rubber (Fig. 1C).

The TorpeDNA is simple to deploy behind any moving vessels, and a single eDNA sample can be collected within just 5 to 10 min of towing and immediately stored in a DNA isolation buffer with no specialized training required (watch video tutorial at https://www.youtube.com/watch?v=9k-31_44THk&t=1s). The simple four-step protocol is user-friendly, making it accessible to both researchers and citizen scientists (Material S1). A detailed list of TorpeDNA components and material types are provided in Table S1. Total cost for all components of one device is estimated at 500 USD. Information on kit production options and associated costs can be obtained *via* the corresponding author, and CAD/3D-printing design files may be shared on a case-by-case basis upon reasonable request, subject to institutional approval and version control considerations.

### Case studies overview

**Figure 2:**
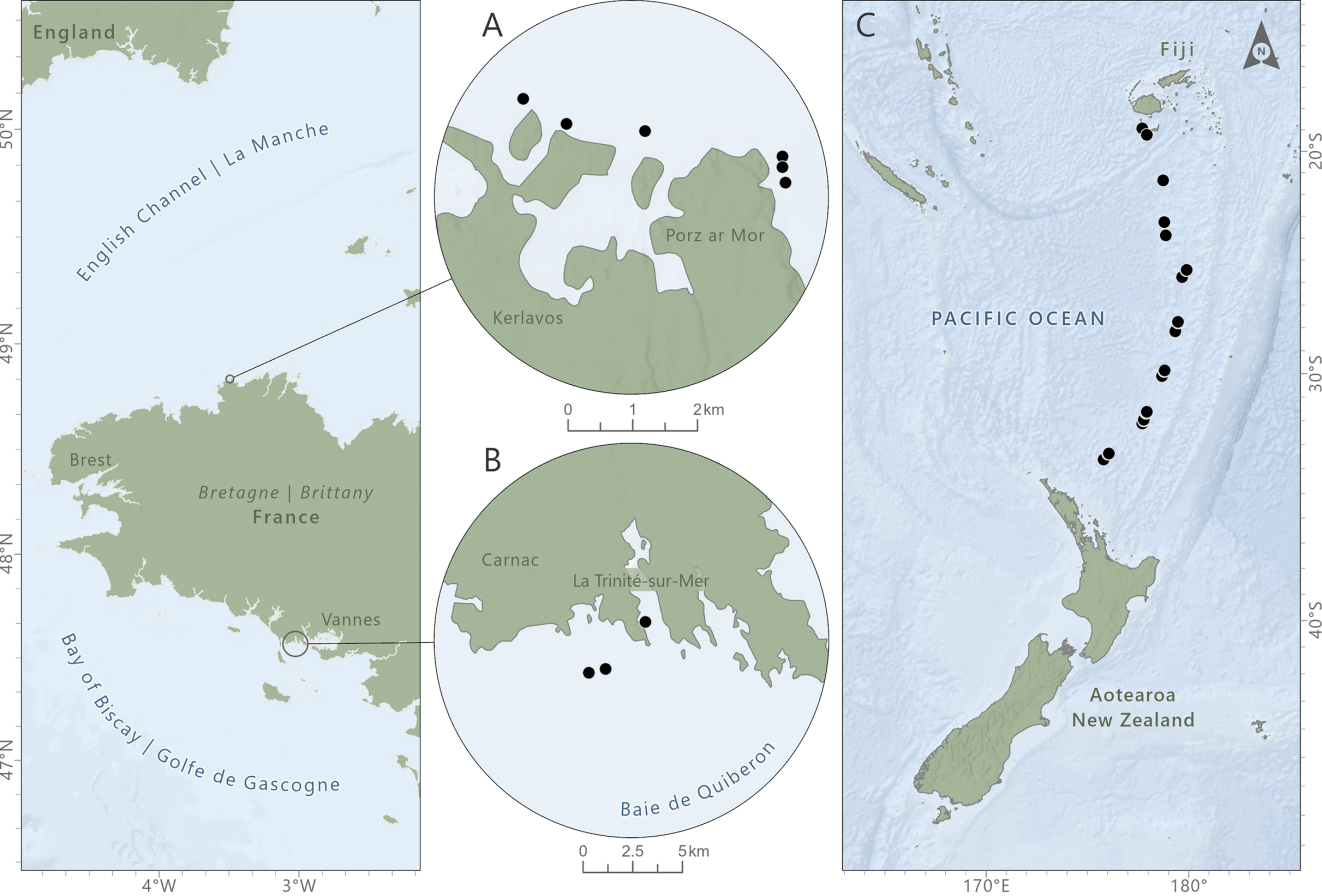
Map showing the study sites for all three marine eDNA case studies in France and the Pacific. (A) Case study 1 in the English Channel, Northern Brittany, France; (B) case study 2 in the Atlantic coast, Bay of Quiberon, France; and (C) case study 3 between Aotearoa-New Zealand and Fiji, in the South-West Pacific. Additional details for each collection station (black dots) can be found in Tables 1 and S2.

To evaluate the versatility and performance of the TorpeDNA device, we conducted three case studies spanning diverse ecological contexts, user profiles, and needs. These case studies were designed to assess the device’s functionality in real-world scenarios, ranging from professionally led research campaigns to participatory citizen science efforts. Each case study examined TorpeDNA’s ability to recover eDNA signals from different biological compartments and environments, and compared its outputs with those of conventional sampling methods. The studies collectively cover a spectrum of marine research domains, from microbial to metazoan ecology and from semi-enclosed basins to continental shelf waters and the high seas (Fig. 2). Sampling strategies included side-by-side comparisons with serial and high-capacity capsule filtration methods, as well as deployments across different vessel types. Different isolation buffers were used across case studies, and a distinct DNA extraction method was applied in case study 1, reflecting site-specific logistical constraints and adherence to partner laboratory protocols; however, downstream PCR amplification and bioinformatic quality-control procedures were standardized at the Cawthron Institute to ensure comparability across datasets. Marine scientific research permits for the collection of seawater filtrates were secured for the Exclusive Economic Zones of Fiji (Collection permit #TPN 546/2025 delivered by the Ministry of Foreign Affairs of the Government of Fiji) and New Zealand (Collection permit #SP822 delivered by the New Zealand Ministry for Primary Industries). In the Exclusive Economic Zone of France, permits are not required for the collection of filtered seawater by a French organization outside of a marine reserve.

**Table 1:** Description of replicated samples from all case studies.

Case study	Experiment	Metabarcode(s)	Sample point	Filter	Samples replicate	Date	Lat	Long
1	TorpeDNA *vs* Waterra	18S (euk), COI (met), 16S (vert)	1	Waterra	2	14/09/24	48.841	−3.503
			1	TorpeDNA	2	14/09/24	48.841	−3.501
			2	Waterra	2	14/09/24	48.838	−3.473
			2	TorpeDNA	2	14/09/24	48.840	−3.472
			3	Waterra	2	15/09/24	48.840	−3.492
			3	TorpeDNA	2	15/09/24	48.840	−3.492
			4	Waterra	2	15/09/24	48.837	−3.473
			4	TorpeDNA	2	15/09/24	48.837	−3.473
			5	Waterra	2	16/09/24	48.843	−3.509
			5	TorpeDNA	2	16/09/24	48.843	−3.509
			6	Waterra	2	16/09/24	48.836	−3.473
			6	TorpeDNA	2	16/09/24	48.836	−3.473
2	TorpeDNA *vs* serial filtration	16S (bac)	P1	TorpeDNA	3	4/02/25	47.573	−3.019
			P1	Ultrafiltration	3	4/02/25	47.573	−3.019
			P2	TorpeDNA	3	4/02/25	47.558	−3.037
			P2	Ultrafiltration	3	4/02/25	47.558	−3.037
			P3	TorpeDNA	3	4/02/25	47.557	−3.045
			P3	Ultrafiltration	3	4/02/25	47.557	−3.045
3	TorpeDNA—open ocean	16S (bac), 18S (euk), COI (met) 16S (vert)	Day 1	TorpeDNA	6	11/05/24	−33.644	175.764
			Day 2	TorpeDNA	9	12/05/24	−32.144	177.693
			Day 3	TorpeDNA	6	13/05/24	−30.115	178.676
			Day 4	TorpeDNA	6	14/05/24	−28.207	179.327
			Day 5	TorpeDNA	6	15/05/24	−25.764	179.680
			Day 6	TorpeDNA	3	16/05/24	−23.306	178.793
			Day 7	TorpeDNA	3	17/05/24	−21.372	178.737
			Day 8	TorpeDNA	9	18/05/24	−18.958	177.733

**Note:**

Complete list, including all latitude-longitude coordinates are available in Table S2. Abbreviations: bac, bacteria; euk, eukaryotes; met, metazoans; vert, vertebrates.

### Samples collection and DNA extractions

#### Case study 1: comparison of TorpeDNA (20 ${\mu}$m filter) and pump filtration (0.45 ${\mu}$m filter)

This case study assessed the performance of the TorpeDNA device relative to a conventional pumping method that uses Waterra™ 0.45 μm eDNA filtration capsules (hereafter referred to as ‘Waterra’), for characterizing coastal eukaryotic communities in Northern Brittany, France. By comparing DNA yield, taxonomic coverage, and community structure across multiple genetic markers, the objective was to evaluate whether TorpeDNA can provide results comparable to conventional fine-pore filtration while offering a more practical and scalable field-deployable alternative for marine biodiversity monitoring.

Sampling was conducted from 14 to 16 September 2024 along the coastal waters of the rocky shores of Northern Brittany, France, using a rigid-hulled inflatable boat (Fig. 2A; Tables 1, S2). Six sampling sites were randomly selected along Trégastel’s coastline. Sub-surface eDNA samples were collected at each site in duplicates using TorpeDNA (47 mm diameter and 20 μm mesh nylon filter; Merck Millipore, Germany) and Waterra™ (600 cm^2^ of polyethersulfone (PES) and 0.45 μm pore filter capsules; Waterra Pumps Limited, Mississauga, Canada) to compare their efficiency in characterizing marine eukaryotic communities. Waterra filtration involved simultaneous filtering of two samples per site for 30 min using a ‘home-made’ pump. Filters were then emptied, and preserved in 50 mL of CTAB lysis buffer, which was stored at 4 °C until DNA extraction. Two TorpeDNA replicates were collected sequentially, with the boat following a circular track around each site. The TorpeDNA was towed 15 m behind the boat for 5 min and at a constant speed of five knots. Between each filtration, the O-ring and the filter holder grid were cleaned with a diluted bleach solution to remove residual DNA, and the torpedo was rinsed for a few seconds by drawing seawater from the sampling site before inserting new filters. TorpeDNA filters were carefully removed using gloves and sterile forceps, immediately placed in tubes containing 1 mL of CTAB lysis buffer and transported on ice to the lab for DNA extraction.

DNA extraction for both TorpeDNA and Waterra filters was performed following a PCI (Phenol-Chloroform-Isoamyl) procedure with isopropyl alcohol precipitation at −20 °C (Renshaw et al., 2015). All samples were incubated overnight at 60 °C in a shaker at 400 rpm. For TorpeDNA, two extraction replicates were performed, each using 700 µL of lysate sample. To each tube, 350 µL of NaCl 4.5M were added and mixed by inversion, before adding 700 µL of chloroform:isoamyl (24:1) (hereafter CI). After inversion, tubes were gently inverted and centrifuged (3 min, 7,400 g). The aqueous layer (supernatant) was transferred to a new tube and mixed with an equal volume of ice-cold isopropanol. The tubes were inverted and stored at −20 °C for at least 1 h, then centrifuged (20 min, 14.000 g). The resulting pellet supernatant was removed, the pellet resuspended and washed in 150 µL of 70% ethanol, and centrifugated again (15 min, 14.000 g). After removing supernatant, the tubes were dried at 60 °C. Both extraction replicates were then pooled by resuspending the DNA pellets in a single 100 µL aliquot of TE buffer. DNA extracts were stored at −20 °C until further processing. For Waterra, starting from 10 mL of CTAB-preserved Waterra sample, DNA extraction was performed in a 50 mL Falcon tube using a scaled-up version of the modified PCI protocol, with 7.5 mL of 4.5M NaCL and 15 mL of CI to separate DNA from proteins and other cellular contaminants. The resulting aqueous phase (~20 mL) was combined with equal volume of ice-cold isopropanol, precipitated at −20 °C for at least 1–2 h. DNA pelleting, washing with 70% ethanol, drying, and resuspension in TE buffer were performed as described for TorpeDNA.

#### Case study 2: comparison of TorpeDNA (20 ${\mu}$m filter) and serial filtration (10 to 0.45 ${\mu}$m fraction)

This case study tested the ability of TorpeDNA to recover bacterial community composition in comparison with a laboratory-based serial filtration approach along a coastal–offshore gradient in Quiberon Bay, France. The experiment aimed to determine how particle-size selectivity influences microbial diversity profiles and to assess whether TorpeDNA can effectively capture the full spectrum of bacterial assemblages, from free-living to particle-associated taxa, under realistic coastal field conditions.

Sampling was conducted on 4 February 2025 at three environmentally and hydrodynamically contrasting sites within Quiberon Bay on the Atlantic coast of South Brittany, France (Fig. 2B; Tables 1, S2, Fig. S1). The first site (P1) was located within the Crac’h River estuary, near an active *Magallana gigas* oyster farming concession (Fig. S1A). The other two sites, P2 and P3, were positioned offshore at the vicinity of treated effluent discharge from the Carnac wastewater treatment plant (WWTP). These locations were chosen based on previous data indicating a recurrent prevalence of norovirus (GI and GII) in oysters between 2020 and 2023 (Ifremer internal reports, IDs 100182 and 95115). During sampling, tidal currents were northerly and low (<0.2 m·s^−1^), placing P2 and P3 directly under the WWTP effluent plume (https://marc.ifremer.fr/). Cumulative rainfall recorded at the nearby Auray station indicated significant precipitation in the week preceding sampling (approximately 120 mm of rainfall; Météo France: https://portail-api.meteofrance.fr/web/fr/), although no raw sewage discharge was officially reported during this period.

At each site, triplicate samples were collected using both TorpeDNA and laboratory-based filtration methods. The TorpeDNA device (20 μm nylon mesh filter; Merck Millipore, Darmstadt, Germany) was towed three times for 5 min at a constant speed of 5 knots, approximately 15 m behind the semi-rigid boat, navigating circularly around the sampling point. Prior to deployment, all sampling components, including the cod-end, O-ring seal, stainless steel grid, 5 L canisters, and forceps, were sterilized in 10% H_2_O_2_ for 1 h and rinsed with sterile distilled water before being stored in aluminium foil. Between each sampling site, the TorpeDNA was cleaned with 10% H_2_O_2_ for 20 min and rinsed with sterile water. Filters were handled with sterile forceps, placed into 2 mL tubes containing RNAlater, and stored at 4 °C until laboratory processing. For the filtration method, 3 L of water were collected per site using a single sterilized canister per site. In the laboratory, water samples were pre-filtered through a decreasing series of nylon mesh (60, 45, 20, and 10 μm), followed by final filtration at 0.45 μm using polyethersulfone (PES) membrane filters (Merck Millipore, Darmstadt, Germany) using a vacuum pump system (Merck Millipore, Darmstadt, Germany; Fig. S1B). Each filtration series was conducted in triplicate (750 mL per replicate), and the resulting 0.45 μm filters were preserved in RNAlater and stored at 4 °C prior to DNA extraction. DNA from each filter was extracted using Qiagen’s PowerSoil Pro Kit on a Qiacube HT system (Qiagen, Hilden, Germany), as per manufacturer’s protocols. An extraction control was included.

#### Case study 3: open ocean sampling with the citizens of the sea initiative

This case study evaluated the applicability of the TorpeDNA device for capturing plankton diversity in challenging open-ocean environments, assessing its performance under real sailing conditions and its potential for large-scale deployment across citizen-science fleets. By analyzing eDNA collected along a 2,000 km transect between New Zealand and Fiji, we aimed to test the device’s ability to recover representative microbial, phytoplankton, and zooplankton communities across broad environmental gradients while demonstrating its scalability for ocean-wide biodiversity monitoring.

Between 11 and 18 May 2024, surface open ocean eDNA was collected across 16 offshore stations during a northbound voyage from Aotearoa New Zealand to Fiji aboard the sailing vessel Love Machine (Fig. 2D; Tables 1, S2). This effort was part of a broader citizen science mission led by the *Citizens of the Sea* initiative (www.citizensofthesea.org), involving 26 yachts equipped with TorpeDNA samplers across the South-West Pacific. However, this case study focuses solely on samples collected from a single vessel to illustrate the field utility and performance of TorpeDNA for open ocean biodiversity monitoring (complete study will be described elsewhere). Stations were selected along a transect spanning over 10 degrees of latitude and 1,737 nautical miles, crossing a wide gradient in sea surface temperature and oceanographic conditions.

At each station, three sub-surface water samples were collected using a single TorpeDNA device (20 μm nylon mesh, 47 mm diameter; Merck Millipore, Darmstadt, Germany), which was deployed sequentially for three consecutive tows. The device was towed approximately 15 m behind the vessel at a speed of 5–12 knots for 5 min per replicate, in accordance with the *Citizens of the Sea* sampling protocol (Material S2). Immediately after recovery, filters were carefully removed using gloves and sterile forceps, stored in 2 mL cryovials containing PowerProtect™ isolation buffer (Qiagen, Hilden, Germany), barcode-scanned using the off the shelf ArcGIS Survey 123 App (Esri, Redlands, CA, USA) for automatic geo-localization, then kept at 4 °C aboard the vessel until arrival onshore. Samples were shipped at chilled temperature to the Cawthron Institute, New Zealand, for further processing. DNA from each filter was extracted using Qiagen’s PowerSoil Pro Kit on a Qiacube HT system (Qiagen, Hilden, Germany), as per manufacturer’s protocols. A blank filter sample was included during extraction.

### Polymerase chain reaction and sequencing analyses

DNA extracts from each case study were PCR amplified using one or more of the following four primer sets targeting bacterial, eukaryotic, metazoan and vertebrate communities. For all case studies, a single PCR reaction was performed per sample and per marker. Case study 1 used the eukaryotic nuclear ribosomal 18S (18S rRNA) Uni18SF and Uni18SR primers (Zhan et al., 2013), targeting a 450 bp fragment of the V4 region, the mitochondrial *Cytochrome oxidase subunit 1* (COI) mlCOIintF and jgHCO2198 (Leray et al., 2013), targeting a 330 bp fragment, and the vertebrate-specific mitochondrial 16S (mt16S rRNA) MarVer3F and MarVer3R primers (Valsecchi et al., 2020), targeting a 245 bp fragment. Case study 2 only used the bacterial ribosomal 16S (16S rRNA) 341F and 805R primer sets (Herlemann et al., 2011; Klindworth et al., 2013), targeting a 470 bp fragment of the V3–V4 region. Case study 3 targeted the bacterial 16S rRNA, the eukaryotic 18S rRNA, metazoan COI, and vertebrate mt16S rRNA (MarVer3) genes, as described above. All primer sets were modified with Illumina overhang adapters to allow library preparation. Polymerase chain reactions (PCRs) were performed using MyFi™ Mix 2× Master Mix (Bioline, London, UK) with 1 μL of each primer (10 μM) and 11 μL of DNA-free water, in a total reaction volume of 30 μL. Paired-end amplicon sizes were approximately 450 bp (16S, 18S, and COI), and 250 (MarVer3). Primer sequences and thermocycling conditions for each primer sets are detailed in Table S2.

Amplification products were normalized using SequalPrep™ Normalization Plates (Thermo Fisher Scientific, Waltham, MA, USA), dual-indexed with Nextera XT adapters, and pooled. Paired-end sequencing was performed at Sequench ltd (Nelson, New Zealand) using V2 chemistry on an NextSeq™ 2000 platform (Illumina, San Diego, CA, USA). Extraction and PCR blanks were included throughout. All raw sequence data were submitted to the NCBI Sequence Read Archive under BioProject ID PRJNA1364753 (active link: https://dataview.ncbi.nlm.nih.gov/object/PRJNA1364753?reviewer=c2k16lehsddmvrknbvkf94jh9h).

### Bioinformatics and biostatistics analyses

#### Bioinformatics pipeline

All fastq files were demultiplexed and their primers removed using CUTADAPT (version 4.9; Martin, 2011), with the default −e value of 0.1, allowing no insertion/deletion and requiring a minimum overlap of 15 bp. Sequences were truncated on their 3′ end at 215 and 190 bp for 16S, and 225 and 226 bp for 18S and COI for the forward and reverse reads, respectively, to remove low-quality calls. Sequence quality filtering and denoising was performed with the DADA2 R package (version 1.30; Callahan et al., 2016), and paired-end reads merged using a minimum overlap of 10 bp. Chimeric sequences were identified and removed using the consensus option of DADA2.

Taxonomic assignation for 16S was performed using the DADA2’s implementation of the RDP Naïve Bayesian Classifier algorithm (Wang et al., 2007) with default settings against the SILVA database (version 138.2; Quast et al., 2012). For 18S, sequences were assigned with the RDP Naïve Bayesian Classifier algorithm applied on SILVA (version 132; Quast et al., 2012) and the PR2 (version 5.0.0; Guillou et al., 2013) databases. For COI, assignments were made using the classification trees (‘insect’) classifier (version 1.4.0.9; Wilkinson et al., 2018) trained on the MIDORI UNIQUE 20180221 training set and approximately 14,000 non-metazoan COI sequences from GenBank (Benson et al., 2018). For MarVer3, 18S and COI, taxonomy was also assigned using blastn and megablast (Camacho et al., 2020) on the GenBank nucleotide database (Benson et al., 2018), using the ‘blastn_taxo_assignment’ function of the biohelper R package (version 0.0.19; https://github.com/olar785/biohelper; see parameters for each gene in Material S3). For each gene, results from different assignment approaches were combined using the ‘taxo_merge’ function of biohelper which first normalizes the taxonomy across the different outputs using the National Center for Biotechnology Information (NCBI) curated taxonomic database (Schoch et al., 2020), and then uses the highest resolution between approaches whenever there is a majority consensus (>50%) across all assigned ranks. At each specific rank, taxonomy is only assigned if there is a majority agreement between methods.

Nuclear mitochondrial DNA sequences (NUMTs) in the COI data were identified and removed using MetaMate (Andújar et al., 2021) and the default specification file. The filtering parameters were chosen to maximize retention of verified authentic sequences (highest recall) while removing as many verified non-authentic sequences (highest accuracy). Amplicon sequence variants (ASVs) were then assigned to operational taxonomic units (OTUs) based on sequence similarity (97%) using the ‘otu’ function of the kmer R package (version 1.1.2; https://github.com/shaunpwilkinson/kmer).

Potential contamination was investigated using negative controls and removed using default settings of the microDecon R package (version 1.0.2; McKnight et al., 2019). Sequencing depth and diversity coverage per sample were investigated using the ‘ggrare’ function of the ranacapa R package (version 0.1.0; Kandlikar et al., 2018). Samples with fewer than 1,000 reads were discarded prior to downstream analysis. For the bacterial 16S dataset, amplicon sequence variants with >10 reads in at least 1 sample were retained, to minimize the presence of potentially spurious sequences. For COI and 18S datasets, amplicon sequence variants with >2 reads in at least 1 sample were retained. For the MarVer3 amplicon, given the inherent scarce nature of vertebrate detections, no ASV filters were applied.

#### Statistical analyses

We used custom R scripts to obtain summary metrics of the data (stored as phyloseq objects) and generate figures. For each of the case studies, we explored alpha and beta diversity patterns and compared how these varied among methods (case studies 1 and 2) or in space (case study 3). For case studies one and two, we estimated ASV richness per sample and performed non-parametric Kruskal-Wallis rank sum tests to evaluate if ASV richness differed between the methods. We also extracted ASV tables from phyloseq objects and generated ASV accumulation curves using the specaccum function from the vegan package (Oksanen et al., 2019), with the “random” method and 100 permutations. We explored patterns of community composition and structure. For all case studies, we estimated the average relative abundance (proportion of reads) of each taxonomic group at the Phylum level for each sample. We used the package microeco (Liu et al., 2021) to estimate relative abundances and produce the corresponding barplots. We also explored community structure by performing principal component analyses (PCA). For each case study, a centered log-ratio (CLR) transformation was applied to the ASV abundance matrix using the function transform from the microbiome R package (Lahti et al., 2017) to account for the compositional nature of the sequence data. Subsequently, a PCA was performed on the CLR-transformed ASV proportion matrix using the prcomp R function (stats package v4.3.3) with centre = true and scale = false options, as the CLR data already addresses the scaling of data. The first two axes were used for visualization using ggplot2 custom scripts. We tested if the sampling method had a significant effect on community composition *via* a PERMANOVA analysis. For this, we calculated a Euclidean distance matrix from the CLR-transformed ASV table and used the adonis2 function with 999 permutations and sample type as a factor. To ensure that observed differences between methods were not simply due to variation in within-group dispersion (*i.e*., differences in the spread or variability of samples within each sampling method), we assessed homogeneity of dispersion using the betadisper and permutest functions (with 999 permutations) from the vegan package. All codes are available in Material S4.

## Results

Across all three case studies, sequencing generated a total of over 84 million raw reads, comprising 17.4 M reads for case study 1 (TorpeDNA *vs* Watera), 8.1 M for case study 2 (TorpeDNA *vs* serial filtration), and 59 M for case study 3 (TorpeDNA—open ocean). Following quality filtering and processing, 48.1 M reads were retained, including 9 M reads for case study 1, 3.5 M for case study 2, and 35.6 M for case study 3. In case study 1, the mean number of reads per sample after filtering was 143,347 for COI, 221,978 for 18S rRNA, and 21,367 for MarVer3. For case study 2, the average post-filtering read count per sample was 195,505. In case study 3, average read counts per sample were 286,269 for 16S rRNA, 256,045 for COI, 247,259 for 18S rRNA, and 97,695 for MarVer3. A summary of read counts is provided in Table S2.

### Case study 1

#### Alpha diversity and sequencing performance comparison

**Figure 3:**
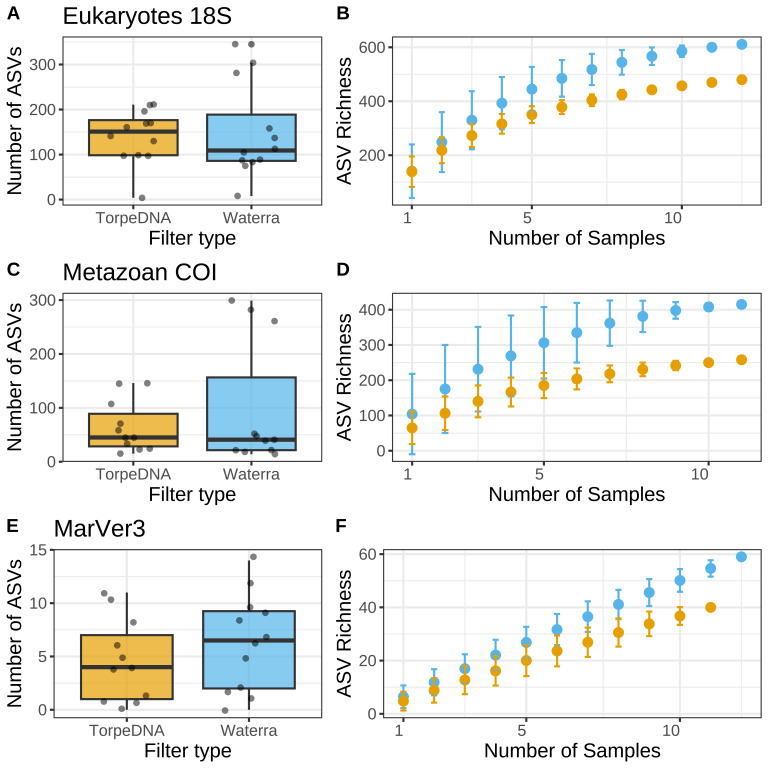
Comparison of amplicon sequence variants (ASVs) richness and accumulation patterns between TorpeDNA and Waterra filtration methods (case study 1). (A, C, E) Boxplots comparing the median and interquartile ranges for the number of amplicons sequence variants (ASVs) between the evaluated filter types. ASV counts per sample are shown as points. (B, D, F) ASV accumulation curves for each filter type, color codes are the same as in left panels. Points indicate mean number of ASVs and vertical bars indicate standard deviations around the mean derived from 500 permutations.

Across all three metabarcodes tested (18S rRNA, COI, and MarVer3), the number of Amplicon Sequence Variants (ASVs) retrieved per sample (alpha diversity), summarized across the 12 samples collected for each method, did not differ significantly between TorpeDNA and Waterra (K-W tests. 18S: X^2^_1df_ = 0.48, *p* = 0.488; COI: X^2^_1df_ = 0.08, *p* = 0.767; MarVer3: X^2^_1df_ = 0.977, *p* = 0.322), despite substantial within-method dispersion among samples and among markers variation (Fig. 3). For the 18S rRNA gene, the mean number of ASVs detected per sample were 145 ASVs with the Waterra filtration method (min = 8, max = 345), and 140 for the TorpeDNA (min = 4, max = 211), with Waterra yielding slightly lower (but not significant) median values but higher dispersion (Fig. 3A). A similar pattern was observed for the COI dataset, where 100 ASVs were recovered on average per sample with Waterra (min = 14, max = 299), and 65 with TorpeDNA (min = 15, max = 146). Median ASV counts were comparable, but again Waterra showed higher dispersion (Fig. 3C). In contrast, the vertebrate-specific MarVer3 marker generated very low richness overall, with a mean of 6 ASVs per sample for Waterra (min = 0, max = 14), and 4.6 for TorpeDNA (min = 0, max = 11), with no clear difference between the two sampling methods (Fig. 3E).

Rarefaction analyses showed that ASV richness accumulated more quickly with increasing sample number in the Waterra samples than in TorpeDNA samples across all markers. Whereas the tests above compare per-sample richness distributions (alpha diversity) between methods, these accumulation curves evaluate cumulative richness across the dataset as additional samples are added, and show that the Waterra method yielded significantly higher ASV richness than TorpeDNA, with the difference becoming increasingly pronounced as sample numbers increased from six or more for the COI and 18S rRNA markers and from nine or more for MarVer3 (Fig. 3). For the 18S rRNA and COI markers, the ASV richness was close to reaching an asymptote for both methods at around eleven samples (Figs. 3B, 3D). By contrast, the vertebrate MarVer3 marker failed to stabilize for either methods (Fig. 3F), indicating that additional sampling effort or the use of alternative markers would be required to fully capture vertebrate diversity.

#### Community composition and structure

**Figure 4:**
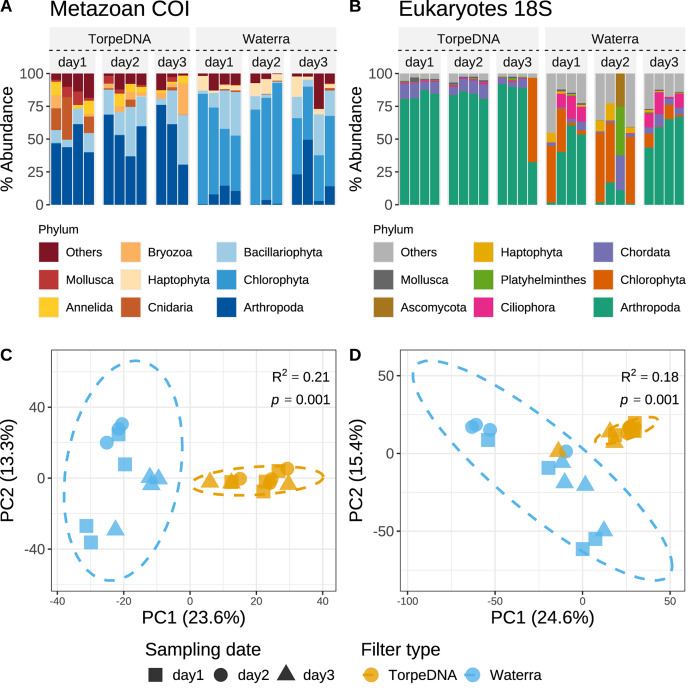
Relative abundance patterns and ordination analyses comparing TorpeDNA and Waterra eukaryotic communities (case study 1). (A, B) Relative abundance of the eight most abundant phyla for the 18S and COI metabarcodes. The remaining phyla were grouped as ‘others’ for simplicity. (C, D) Show the results of Principal Component Analysis for each metabarcode performed on the centred log-ratio-transformed Amplicon Sequence Variant (ASV) abundance data. Each point represents a sample, coloured by the filter type. Shapes indicate different sampling dates. Ellipses encompassing 95% of the data points are displayed with dashed lines. PERMANOVA R^2^ and *p* values are shown at the top right corner of each plot.

Taxonomic profiles derived from the 18S rRNA and COI markers revealed significant differences in community composition between the two filtration methods (PERMANOVA results: 18S Pseudo-F = 4.87, *p* = 0.001; COI pseudo-F = 5.47, *p* = 0.001). Dispersion was not significantly different between filtration methods for COI (Pseudo-F = 0.027, *p* = 0.879), but did differ among them for the 18S (Pseudo-F = 16.31, *p* = 0.001). At the phylum level, TorpeDNA recovered relatively consistent proportions of dominant groups across replicates and sampling days and favored Animalia taxa, with Arthropoda and Chordata prevailing in the 18S rRNA dataset, while COI amplicons were more temporally variable and dominated by Arthropoda, Chlorophyta, and other metazoan lineages (Figs. 4A, 4B). In contrast, the Waterra method produced higher variability both among replicates and across sampling days and was more in favor of protists despite a good coverage of Arthropoda. Dominant phyla in the Waterra samples included Arthropoda, Chlorophyta, and Ciliophora in the 18S rRNA dataset (Fig. 4B), and Chlorophyta, Bacillariophyta (diatoms), and Arthropoda in the COI dataset (Fig. 4A). Minor differences were also evident in the relative abundance of less common phyla, particularly in the 18S rRNA dataset where Waterra occasionally retrieved higher proportions of rare taxa.

Ordination analyses based on centred log-ratio–transformed ASV abundances revealed a consistent clustering of TorpeDNA samples across replicates and sampling days for both the 18S and COI markers (Figs. 4C, 4D). The one outlier sample on day 3 had a low sequence count (*n* = 2,294 reads) just above the set 1,000 reads threshold, accounting for the discrepancies shown on Figs. 4B, 4D. In contrast, communities recovered with the Waterra method displayed greater dispersion in ordination space, indicating higher heterogeneity among replicates and across sampling dates (Figs. 4C, 4D).

The diversity of marine vertebrates detected with the MarVer3 marker was extremely low and sporadic across both sampling methods (Fig. S2A). The most frequently recovered ASVs corresponded to terrestrial sources such as humans, pigs, chickens, and dogs, alongside a few local marine taxa including seabream (*Pagrus* sp.). Additional species such as horse mackerel (*Trachurus* sp.), wrasse (*Labrus* sp.), and sardines (*Sardina* sp.) were detected only occasionally in the Waterra samples, with occurrences ranging from 1 to 6 samples. Wrasses are coastal rocky reef dwellers (Quignard & Pras, 1986) while seabreams are also coastal inhabitants, but found on a larger range of habitats (Bauchot & Hureau, 1986). In contrast, horse mackerel and sardine are more widely distributed, benthopelagic for the former (Smith-Vaniz, 1986) and pelagic for the later (Whitehead, 1986). Thus, surface samples detected both bottom dwelling and pelagic species.

### Case study 2

#### Alpha diversity, community composition and structure

**Figure 5:**
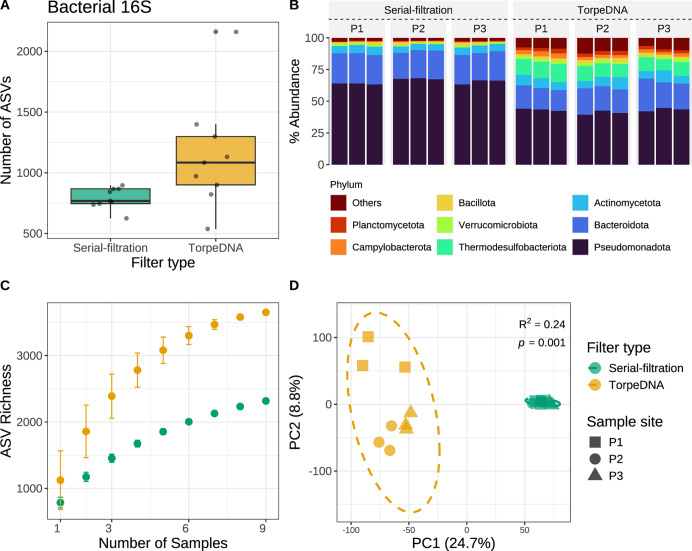
Bacterial diversity, composition, and multivariate structure comparing TorpeDNA and serial filtration (case study 2). (A) Boxplots showing the median and interquartile ranges for the number of Amplicon Sequence Variants (ASVs) between the evaluated filter types. (B) Barplot displaying the relative abundance of the eight most abundant phyla for the 16S metabarcode. (C) ASV accumulation curves for each filter type, color codes are the same as in (A). (D) Results of Principal Component Analysis performed on the centred log-ratio-transformed ASV abundance data. Each point represents a sample, coloured by the Filter type. Shapes indicate different sampling sites. Ellipses encompassing 95% of the data points are displayed with dashed lines. PERMANOVA R^2^ and *p* values are shown at the top right corner of each plot.

For the bacterial 16S rRNA marker, both TorpeDNA and serial-filtration approaches recovered high levels of diversity, though the TorpeDNA yielded significantly higher numbers of ASVs (mean 1,200) compared with serial-filtration (mean 750) (K-W test. X^2^_1df_ = 5.902, *p* = 0.015) (Fig. 5A), which was also evident on the richness accumulation curve (Fig. 5C).

Barplots of the eight most abundant bacterial phyla (Fig. 5B) showed that both sampling methods consistently detected the same dominant groups across sites, with clear differences in proportional representation. Serial filtration samples were strongly dominated by *Pseudomonadota* and *Bacteroidota*, which together comprised the large majority of reads. In contrast, although TorpeDNA samples were also dominated by these two phyla, their relative contributions were lower, and additional phyla, such as *Thermodesulfobacteriota* and *Actinomycetota*, contributed more substantially to overall community composition.

Principal component analysis showed clear method-dependent patterns. Serial filtration replicates formed one tight single cluster, including all sites (P1–P3), whereas TorpeDNA samples also grouped by site but displayed greater within-site dispersion (Fig. 5D). PERMANOVA analyses revealed a significant effect of filtering method on community structure (Pseudo-F = 5.12, *p* = 0.001), and the betadisper analysis revealed significant differences in dispersion between filtration methods (Pseudo-F = 454.1, *p* = 0.001).

#### Shared bacterial taxa

**Figure 6:**
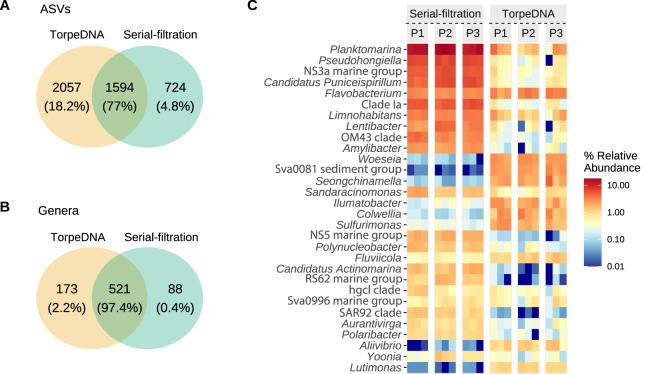
Comparative diversity and dominant bacterial genera recovered by TorpeDNA *vs* serial filtration (case study 2). (A) Venn diagram showing the number of ASVs unique to each method and common to both as part of case study 2 (TorpeDNA *vs* Serial filtration). In parentheses, the percentage of reads that fall in each category. (B) Venn diagram showing the number of bacterial genera shared and unique to each filtration method. In parentheses, the percentage of reads that fall in each category. (C) Heatmap displaying the relative abundance of the top 30 most abundant bacterial genera.

Comparison of ASV and genus-level composition revealed that TorpeDNA and serial filtration recovered substantially overlapping bacterial communities, yet with notable fractions of non-shared taxa, particularly with TorpeDNA (Figs. 6A, 6B). A total of 1,594 ASVs were shared between the two methods, representing 77% of total sequence reads, indicating strong agreement in dominant bacterial groups (Fig. 6A). However, each method also retrieved a distinct subset of ASVs, reflecting method-specific biases possibly linked to particle size capture. The TorpeDNA captured 2,057 unique ASVs (18.2%), while the serial filtration captured 724 unique ASVs (4.8%). At the genus-level, 521 genera (comprising 97.4% of the reads) were captured by both methods, while 173 (2.2% of reads) and 88 (4.8% of reads) genera were uniquely captured by the TorpeDNA and serial-filtration, respectively.

Heatmap analyses of the 30 most abundant bacterial genera (Fig. 6C) showed clear differences in taxonomic composition between filtration methods. Serial filtration samples were dominated by pelagic-associated genera such as *Pseudohongiella*, *Candidatus Puniceispirillum*, *Lentibacter*, and *Amylibacter*. In contrast, TorpeDNA samples showed higher relative representation of genera commonly associated with particles or sediments, including *Woeseia*, *Seongchinamella*, *Ilumatobacter*, *Colwellia*, and *Sulfurimonas*. Several genera were detected by both methods, including *Planktomarina*, *Flavobacterium*, and *Sandaracinomonas*.

### Case study 3

#### Community composition & structure

Across the latitudinal transect from New Zealand (−34°) to Fiji (−19°), relative abundance of planktonic taxa at the phylum level revealed notable distributional shifts in microbial and metazoan community composition. For the bacterial 16S rRNA dataset (Fig. S3A), *Proteobacteria* dominated throughout but gradually decreased toward tropical stations, where *Cyanobacteria* and *Bacteroidota* became more abundant. The eukaryotic 18S rRNA and metazoan COI datasets (Figs. S3B, S3C) were both largely dominated by Arthropoda and showed more subtle compositional turnover along the transect, with temperate samples enriched in Arthropoda, and tropical samples characterized by increasing representation of Cnidaria, Echinodermata, Chaetognatha and Bacillariophyta. Principal Component Analyses at the ASV level revealed clear latitudinal structuring for all three marker genes (Figs. S4D–S4F), indicating a strong environmental or biogeographical gradient shaping community composition across the South Pacific. The number of ASVs obtained across the New Zealand to Fiji transect was relatively constant for the 16S and 18S rRNA genes, while decreasing slightly for the COI marker (Fig. S4).

**Figure 7:**
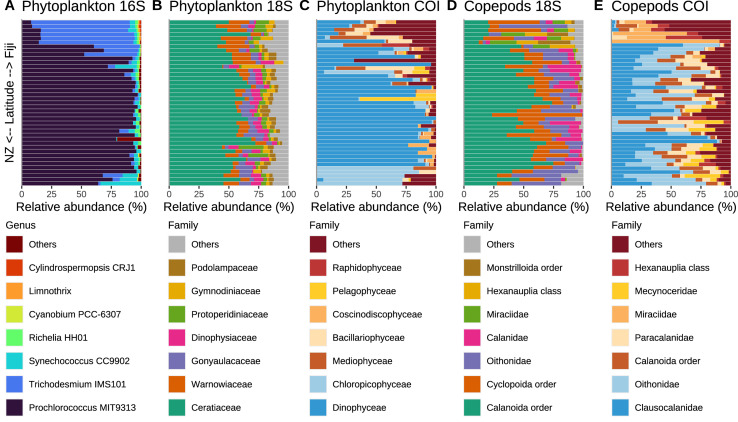
Latitudinal shifts in microbial, phytoplankton, and zooplankton assemblages along the open-ocean transect (case study 3). Relative abundance of the seven most abundant genera for the bacterial 16S rRNA (A), and families of phytoplankton derived from the eukaryotic 18S rRNA (B) and metazoan COI (C) markers, as well as families of crustacean copepods derived from the 18S rRNA (D) and COI (E) markers. The remaining phyla were grouped as ‘others’ for simplicity.

Latitudinal shifts in community composition were detected across all markers from New Zealand to Fiji (Fig. 7). For phytoplankton, bacterial 16S rRNA profiles showed a transition from *Prochlorococcus* and *Synechococcus* to increased representation of *Trichodesmium* at lower latitudes (Fig. 7A). The 18S rRNA dataset was dominated by dinoflagellate families, with Ceratiaceae prevailing across the transect and additional families such as Gonyaulacaceae, Dinophysiaceae, Gymnodiniaceae and Podolampaceae present at varying proportions; Warnowiaceae occurred throughout and increased toward the tropics (Fig. 7B). The COI metabarcode captured a broader range of phytoplankton families, including chlorophytes, dinoflagellates, diatoms and heterokonts, with differences in relative representation between temperate and tropical stations (Fig. 7C). Copepod communities inferred from both 18S rRNA and COI also varied with latitude (Figs. 7D, 7E), with temperate stations characterised by families such as Calanidae, Clausocalanidae and Mecynoceridae, and tropical stations showing higher representation of Miraciidae, Paracalanidae and Oithonidae.

The diversity of vertebrate DNA detected in open-ocean samples using the MarVer3 primer set (Fig. S2B) was low yet provided some information regarding pelagic taxa. The most prevalent amplicon sequence variants (ASVs) corresponded to *Homo sapiens*, indicating the introduction of human DNA contamination during sample collection and/or processing. Beyond these, several marine fish lineages were detected in a low number of samples, including ray-finned fishes (Carangiformes), anchovies (*Encrasicholina* sp.), and multiple lanternfish taxa (Myctophidae, *Hygophum* sp., *Ceratoscopelus* sp.), as well as flying fish (*Cheilopogon* sp.), all characteristic of open-ocean and epipelagic environments.

## Discussion

### Methodological performance and limitations

The three case studies presented here collectively demonstrate that the TorpeDNA device provides an easy-to-use, robust, flexible, and fit-for-purpose alternative to conventional filtration systems for the collection of environmental DNA across diverse marine settings. Despite its coarse mesh size (20 µm) and simplified design, TorpeDNA recovered biodiversity profiles that were comparable to, or even more comprehensive for some target taxa than those obtained through gold-standard filtration approaches such as Waterra (0.45 µm) and serial filtration (10 to 0.45 µm fraction), across microbial, eukaryotic, and metazoan assemblages. These findings indicate that a substantial fraction of eDNA, particularly the cell-bound and particle-associated component, is efficiently captured by the device.

Across all case studies, TorpeDNA’s performance was consistent and reproducible, highlighting the operational simplicity and reliability of its continuous-flow design. The integrated cod-end filtration mechanism allows direct in-water capture without the need for pumps, hoses, or peristaltic systems, which often represent major logistical bottlenecks in offshore operations (Bowers et al., 2021; Patin & Goodwin, 2023). An important yet often underappreciated consideration in developing eDNA sampling technologies is ensuring that instrument design matches the range of user needs and operational contexts. Each system represents a compromise between analytical performance, usability in the field, and affordability, as no single device can satisfy every application or environment (Yamahara et al., 2025). Within this context, TorpeDNA was purposefully designed to prioritize field robustness, user accessibility, and scalability over extreme precision or automation, thereby filling a key niche between laboratory-grade and fully-autonomous systems. In practice, this niche centers on rapid, standardized eDNA collection during routine vessel transits and offshore passages to support baseline mapping and repeated transect monitoring in settings where traditional pump-based or automated systems are impractical. The TorpeDNA can be deployed at cruising speeds of up to 12 knots with a 20 µm mesh size, greatly expanding its operational range compared with earlier prototypes such as the Cruising Speed Net (von Ammon et al., 2020, 2023). This capability enables time-efficient sampling of large sub-surface water volumes. As filtration proceeds, fine particulate material can accumulate on the nylon mesh and partially clog it, effectively reducing pore space and allowing retention of particles smaller than the nominal 20 *µm* size (Pochon et al., 2024). It can be deployed across diverse vessel platforms, from small inflatable boats and yachts to large research or commercial vessels operating in dynamic offshore conditions. Furthermore, its modular, low-cost design and compatibility with standard filter formats make TorpeDNA a practical, scalable, and user-friendly tool for expanding distributed ocean observation networks and supporting environmental monitoring programs requiring minimal training.

Despite these advantages, the comparative analyses of Case Studies 1 and 2 revealed method-specific differences in diversity estimates and community composition. In Case Study 1, both TorpeDNA and Waterra filtration captured broadly similar ASV richness, but the Waterra system showed higher cumulative richness, greater temporal variability, and a greater proportion of phytoplankton taxa such as chlorophytes, diatoms and ciliates. These results likely reflect Waterra’s finer pore size (0.45 µm) and higher retention of single-cell organisms and potentially extracellular DNA fragments (Peixoto et al., 2021; Power et al., 2023; Vautier et al., 2025), suggesting that this approach may be more sensitive to fine-scale micro-patchiness of nano-sized plankton. By contrast, TorpeDNA’s 20 µm mesh preferentially retained larger organisms and particle-attached cells, resulting in a smoother community signal among replicates and sampling days. This reduced micro-patchiness suggests that TorpeDNA integrates over a broader water volume, producing community profiles that are representative at the local-to-regional scale. However, because effective filtered volume was not directly quantified in this study, we cannot exclude the possibility that differences in water volume processed also contributed to the observed between-sample variability. Discrepancies between the two methods therefore reflect inherent differences in pore size and water throughput rather than inconsistencies in molecular processing.

In Case Study 2, both the serial-filtration system and TorpeDNA recovered the same dominant bacterial phyla (*e.g*., *Pseudomonadota*, *Bacteroidota*, and *Actinomycetota*), yet differed in relative abundance patterns and community dispersion. Serial filtration produced tightly clustered assemblages dominated by pelagic, bloom-associated genera such as *Pseudohongiella*, *Candidatus Puniceispirillum*, *Lentibacter*, and *Amylibacter*, taxa commonly enriched in productive surface waters and during phytoplankton blooms (Xu et al., 2019; Lee et al., 2019; Li et al., 2012; Teramoto & Nishijima, 2014). In contrast, TorpeDNA samples showed greater heterogeneity among replicates and a higher representation of particle-associated or sediment-linked genera, including *Woeseia*, a degrader of protein-rich organic matter in marine sediments (Lian et al., 2025); *Ilumatobacter* and *Seongchinamella*, typical of estuarine and tidal-flat environments (Matsumoto et al., 2013; Kang et al., 2020); *Colwellia*, often abundant in cold or sediment-influenced waters (Deming & Junge, 2015); and *Sulfurimonas*, associated with sulfidic micro-niches (Han & Perner, 2015). These contrasts are consistent with the different physical sampling regimes: serial filtration preferentially captures small, free-living cells, whereas TorpeDNA’s larger mesh (20 µm) and turbulent inflow facilitate retention of aggregates and particle-attached microbes, thereby integrating signals from multiple micro-habitats within the water column. Several cosmopolitan genera, including *Planktomarina*, *Flavobacterium*, and *Sandaracinomonas*, were shared across both methods, indicating overlap in core community structure despite method-specific differences in dispersion. Overall, these results support the use of TorpeDNA as a practical screening tool for applications in marine microbiology, including routine microbial surveillance near aquaculture production areas and wastewater-impacted environments (Bouchez et al., 2016), while highlighting that differences between methods are expected outcomes of their respective filtration and size-fraction capture properties rather than analytical artefacts.

A key limitation observed across both Waterra and TorpeDNA methods was the weak detection of vertebrate DNA. Despite the use of the widely applied MarVer3 marker (Valsecchi et al., 2020, 2021; Boyse et al., 2024; Shaffer et al., 2025), vertebrate reads were sparse and dominated by common anthropogenic or domestic sources from terrestrial water runoffs, with few local marine taxa detected. Because both methods yielded similarly poor recoveries, the limitation cannot be attributed to differences in filter pore size, suggesting that other factors are at play. The MarVer3 marker is known to be highly susceptible to human and mammalian contamination (Valsecchi et al., 2020; Boyse et al., 2024), and this was evident in our dataset where preferential amplification of non-marine vertebrates likely overwhelmed the true signal of marine taxa. This unexpected result could stem from multiple, non-exclusive causes, including potentially insufficient volumes of water filtered, the low natural concentrations of vertebrate eDNA in the sampled environments, the DNA extraction protocols employed, and potentially sub-optimal thermocycling parameters for this primer pair. Improving the detection of marine vertebrates and mammals remains an active area of research, and we are currently conducting targeted comparative experiments to resolve this issue (X. Pochon, O. Laroche, E. Bomati, P. Saenz, 2026, in preparation). These include cross-platform assessments of TorpeDNA along coastal-to-offshore gradients, testing alternative filtration pore sizes and isolation buffers, evaluating optimized extraction kits, and applying a broader suite of vertebrate-specific markers such as MarVer1 (Valsecchi et al., 2020), MiFish-U (Miya et al., 2015), Fish16S (Berry et al., 2017; Deagle et al., 2007), and uCeta (Ushio et al., 2025). Ongoing metabarcoding analysis using the MiFish 12S marker on several dozen TorpeDNA samples recently collected around the same site in Northern Brittany identified around 50 species of fish, highlighting the potential of this method for fish inventory (E. Quéméré, T. Urvois, L. Baulier, G. Boussarie, A. Deniau, P. Provost, V. Trenkel, 2026, unpublished data). The integration of these improvements is expected to significantly enhance the recovery of rare vertebrate eDNA and expand the taxonomic reach of TorpeDNA toward higher-trophic-level organisms.

### Ecological and biogeographical insights from multi-marker datasets

Beyond its methodological validation, this study demonstrates TorpeDNA’s capacity to capture ecologically coherent and biogeographically structured biodiversity patterns across a wide range of marine environments. The consistency of community gradients observed across case studies, from coastal microbial transitions to latitudinal plankton turnover in the open ocean, confirms that the device retrieves biologically and ecologically meaningful signals. The strong congruence between molecular patterns and known oceanographic gradients provides compelling evidence of TorpeDNA’s ability to detect the hierarchical organization of marine biodiversity across spatial and trophic scales (Follows et al., 2007; Ladau et al., 2013; Ward, Dutkiewicz & Follows, 2014).

The open-ocean deployment (case study 3) most clearly illustrates this potential and reflects the primary motivation behind the development of TorpeDNA, *i.e*., to provide a fit-for-purpose, citizen-science-ready device enabling reliable eDNA collection in extremely challenging offshore conditions. Designed to be compact, safe, and operable by non-specialists, TorpeDNA was successfully deployed by *Citizens of the Sea* vessels during the 2024 Pacific Rally, a participatory ocean-sampling initiative that involved 26 yachts actively collecting eDNA samples around the South-West Pacific. The results reported here represent only a single vessel from that fleet, yet already reveal clear latitudinal and trophic gradients. The shift in 16S rRNA cyanobacterial signals from *Prochlorococcus* and *Synechococcus* toward *Trichodesmium* is consistent with well-established functional turnover in open-ocean primary producers and nitrogen fixers (Hoffmann, 1999; Rajaneesh et al., 2020; Dwivedi & Ahmad, 2023). Similarly, marker-dependent differences in phytoplankton composition (18S rRNA emphasizing dinoflagellate families, and COI capturing a broader spectrum including chlorophytes, diatoms and heterokonts) likely reflect both ecological structuring and known primer and reference-database biases, while still aligning with basin-scale plankton biogeography driven by temperature, nutrient availability, and water-mass structure (Chisholm, 1992; Flombaum et al., 2013; Bock et al., 2022; de Vargas et al., 2022; O’Rorke et al., 2022). The pronounced turnover in copepod families from calanoid-dominated temperate assemblages to tropical communities enriched in smaller taxa supports the inference of coherent zooplankton biogeographic transitions along this transect. Remarkably, TorpeDNA captured these large-scale ecological transitions with only brief daily sampling (~20 min) and at substantially lower cost than conventional eDNA surveys, underscoring its effectiveness in integrating biodiversity signals across mesoscale gradients.

The use of multiple genetic markers further demonstrated TorpeDNA’s strength in providing a multi-trophic view of ocean life. The combined 16S, 18S, and COI datasets captured complementary components of the microbial, phytoplanktonic, and metazoan assemblages, enabling assessment of trophic coupling and biogeochemical connectivity across scales (Nef, Pierella Karlusich & Bowler, 2024; Zamora-Terol, Novotny & Winder, 2020; Novotny, Zamora-Terol & Winder, 2021; Bucklin et al., 2021). When expanded to a full citizen-science fleet, such multi-marker data collection has the potential to generate spatio-temporal biodiversity datasets at an unprecedented scale, offering new opportunities to track ecosystem change, climate-driven biogeographic shifts, and biodiversity loss across the global ocean (P. Saenz, O. Laroche, B. Knight, E. Bomati, X. Pochon, 2026, in preparation). Collectively, these findings validate TorpeDNA as an ecologically reliable and socially inclusive instrument, bridging professional research and citizen participation to illuminate ocean life across vast and previously unsampled regions.

### Future improvements, scalability and applications

Further refinements are needed to optimize the TorpeDNA’s precision and expand its range of applications. A key next step involves quantifying the water flow rate within the device under different towing conditions to derive accurate estimates of the total volume filtered and, ultimately, eDNA concentration per liter. Computational Fluid Dynamics (CFD) simulations indicate that, given the 14% porosity of the 20 µm filter, flowthrough rates are approximately 12.8 L min^−1^ at 5 knots and 23.9 L min^−1^ at 12 knots (B. Thomsen, 2024, personal communication; Blender Ltd., Auckland, New Zealand). However, these theoretical values may vary *in situ* due to drag forces, turbulence, water current, changes in water turbidity or accumulation of particles on the membrane, all of which can influence both flow rate and DNA retention efficiency (Shogren et al., 2016; Pourbozorg, 2017; Kumar et al., 2022; Obligado & Bourgoin, 2022). Future in-field tests should incorporate compact inline turbine sensors or miniature ultrasonic flowmeters to obtain real-time hydrodynamic data (Nour & Hussain, 2020), thereby improving quantitative interpretations of eDNA yield and biomass accumulation.

TorpeDNA’s modular 3D-printed design facilitates rapid modification and integration of new components. For instance, the cod-end filtration unit is currently being adapted for in-water serial filtration aimed at microbiological applications (Y. Reynaud, X. Pochon, K. Lhaute, 2026, in preparation). In this configuration, the end-cap (component 11; Fig. 1C) is modified to enable the serial assembly of multiple separated filter grid segments, each fitted with a distinct mesh filter down to 0.2 µm. The selected printing polymer withstands repeated autoclave sterilization cycles (90 min), ensuring both decontamination of DNA and microorganisms, and structural durability (Suyama & Kawaharasaki, 2013). This innovation eliminates the need for chemical sterilant such as 10% H_2_O_2_, streamlining field readiness and laboratory preparation. Flow testing and field validation will determine its operational feasibility and influence on DNA recovery efficiency.

The standard 47 mm diameter of the TorpeDNA filter housing can accommodate a broad range of off-the-shelf filter types with varying pore sizes and chemistry. This flexibility opens pathways for testing advanced membranes such as positively charged substrates or hybrid-capture systems (Bessey et al., 2021; Jeunen et al., 2024; Quek & Ng, 2024), which could substantially increase the capture of rare or degraded DNA fragments, including vertebrate and macro-organism traces.

A defining strength of the TorpeDNA lies in its scalability, both geographically and operationally. Its affordability, portability, and compatibility with standard molecular workflows make it ideal for integration into distributed sampling networks. The *Citizens of the Sea* pilot program illustrates this potential. For example, by pairing TorpeDNA sampling with GPS-linked metadata collection, non-specialist operators helped generate high-quality biodiversity data over thousands of kilometers. When replicated across research vessels, sailing routes, and cargo fleets, such an approach could yield a near-real-time global eDNA monitoring network, comparable in reach to the Continuous Plankton Recorder program (Reid et al., 2010), but with far greater taxonomic resolution. Establishing shared standard operating procedures from sample collection to data archiving (Klymus et al., 2024), as well as FAIR/CARE-compliant databases, and interoperable analytical pipelines (Takahashi et al., 2025), will be essential to realize this vision. In addition, TorpeDNA’s coarse-mesh design highlights its strong potential and versatility in highly turbid waters, such as tropical estuaries or rivers, where small-pore filters clog rapidly (Turba, Thai & Jacobs, 2023).

Ultimately, TorpeDNA provides a practical framework for integrating biodiversity monitoring into global ocean-observation systems. By linking multi-trophic molecular data with environmental variables, it can serve as a cost-effective early-warning and validation tool for ecosystem-based management, contributing directly to the goals of the UN Decade of Ocean Science, the Convention on Biological Diversity, and emerging blue-carbon and biodiversity-credit initiatives.

## Conclusion

This study provides the first multi-context evaluation of TorpeDNA as a scalable and inclusive eDNA collection tool for marine biodiversity observation. In line with our research objectives, TorpeDNA (1) recovered taxonomic diversity and community composition patterns that were broadly comparable to established eDNA sampling approaches across contrasting ecological and operational contexts, while highlighting method-dependent differences linked to filtration regimes and target taxa; and (2) demonstrated operational simplicity and robustness compatible with scalable deployment by non-specialists, including citizen-science settings. By combining scientific rigor with broad accessibility, TorpeDNA bridges the gap between high-precision research instruments and operational devices deployable at scale. Across contrasting marine environments, the device yielded reproducible and ecologically meaningful biodiversity profiles while maintaining simplicity, robustness, and affordability. While it may not replace fine-pore filtration for studies requiring maximal molecular recovery or ultra-trace detection, it offers a reliable, fit-for-purpose solution for large-scale biodiversity mapping and temporal monitoring. With continued refinement and standardisation, TorpeDNA could contribute to global, molecularly informed ocean-observation networks, supporting researchers, managers, industries, and citizen scientists in efforts to observe, understand, and protect marine life in a rapidly changing ocean.

## Supplemental Information

10.7717/peerj.21390/supp-1 Supplemental Information 1 Study area and serial filtration setup used in Case Study 2. (**A**) Location of sampling sites in Quiberon Bay (Case Study 2). (**B**) Devices for filtering seawater from 60 µm to 0.45 µm under sterile conditions (Case Study 2).

10.7717/peerj.21390/supp-2 Supplemental Information 2 Heatmap of vertebrate detections using the MarVer3 assay across sampling methods and case studies 1 and 3. Heat map illustrating the detections of different vertebrates (including terrestrial), assigned with the mt16S MarVer3 metabarcode assay, between (**A**) the TorpeDNA and Waterra sampling methods (case study 1), and (**B**) the vertebrate taxa detected across the latitudinal transect of case study 3. For each taxa, red indicates positive detection and blue indicates no detection.

10.7717/peerj.21390/supp-3 Supplemental Information 3 Phylum-level profiles and multivariate structure of 16S rRNA, 18S rRNA, and COI Amplicon Sequence Variants (ASVs) across the open-ocean transect (case study 3). Relative abundance of the 7 most abundant phyla for the bacterial 16S rRNA (**A**), eukaryotic 18S rRNA (**B**) and metazoan COI (**C**) metabarcodes. The remaining phyla were grouped as “others’. (**D**, **E**, **F**) show the results of Principal Component Analysis for each metabarcode performed on the centred log-ratio-transformed amplicon sequence variant (ASV) abundance data, overlayed with colours representing the latitudinal gradient from New Zealand (−34°) to Fiji (−19°).

10.7717/peerj.21390/supp-4 Supplemental Information 4 Latitudinal patterns of Amplicon Sequence Variants (ASVs) richness for 16S rRNA, 18S rRNA, and Cytochrome C Oxidase I (COI) markers. Distribution of the number of ASVs across different latitudes for the 16S rRNA (**A**), 18S rRNA (**B**) and COI (**C**) markers depicted in case study 3.

10.7717/peerj.21390/supp-5 Supplemental Information 5 List of parts and material components of the TorpeDNA device.

10.7717/peerj.21390/supp-6 Supplemental Information 6 (Excel Sheet 1) Complete list of replicated samples from case studies 1-3, including sample types, sample IDs, collection dates, and latitude-longitude coordinates. (Excel Sheet 2) Molecular primers used across case studies, including primer names, target gene, primer sequence, Primer length, Amplicon length, GC content, Annealing temperature, PCR conditions and references. (Excel Sheet 3) Sequence reads summary.

10.7717/peerj.21390/supp-7 Supplemental Information 7 TorpeDNA CFD-Optimized Design. Improved TorpeDNA features obtained from Blender Ltd (Auckland, NZ), following computational fluid dynamics (CFD) analyses in February 2024, which reduced the drag of the device by 55%.

10.7717/peerj.21390/supp-8 Supplemental Information 8 TorpeDNA Field Protocol. Step-by-step protocol used by Citizen Scientists as part of the *Citizens of the Sea* initative for the collection and isolation of eDNA samples using the TorpeDNA device.

10.7717/peerj.21390/supp-9 Supplemental Information 9 Marker-Specific Taxonomic Settings. Parameters for taxonomic assignments (blastn_taxo_assignment function of the biohelper R package) used in this study for the 18S rRNA (18S), Cytochrome C Oxidase I (COI), and mitochondrial 16S (MarVer3) markers.

10.7717/peerj.21390/supp-10 Supplemental Information 10 R code for all statistical analyses and graphical outputs.

## Additional Information and Declarations

### Field Study Permissions

The following information was supplied relating to field study approvals (*i.e*., approving body and any reference numbers):

Marine scientific research permits for the collection of seawater filtrates were secured for the Exclusive Economic Zones of Fiji (Collection permit #TPN 546/2025 delivered by the Ministry of Foreign Affairs of the Government of Fiji) and New Zealand (Collection permit #SP822 delivered by the New Zealand Ministry for Primary Industries). In the Exclusive Economic Zone of France, permits are not required for the collection of filtered seawater by a French organization outside of a marine reserve.

### Data Availability

The following information was supplied regarding data availability:

The code is available in the Supplemental File.

The sequences are available at NCBI: BioProject #PRJNA1364753.
