# Supplementary material for: TorpeDNA: a fit-for-purpose eDNA sampling device for marine biodiversity monitoring across applications and scales
Source: PeerJ. 2026 Jun 22;14:e21390. doi: 10.7717/peerj.21390 (PMC13296811; doi:10.7717/peerj.21390)
Supplement: Supplemental Information 1 — (A) Location of sampling sites in Quiberon Bay (Case Study 2). (B) Devices for filtering seawater from 60 µm to 0.45 µm under sterile conditions (Case Study 2). [file peerj-14-21390-s001.pdf]

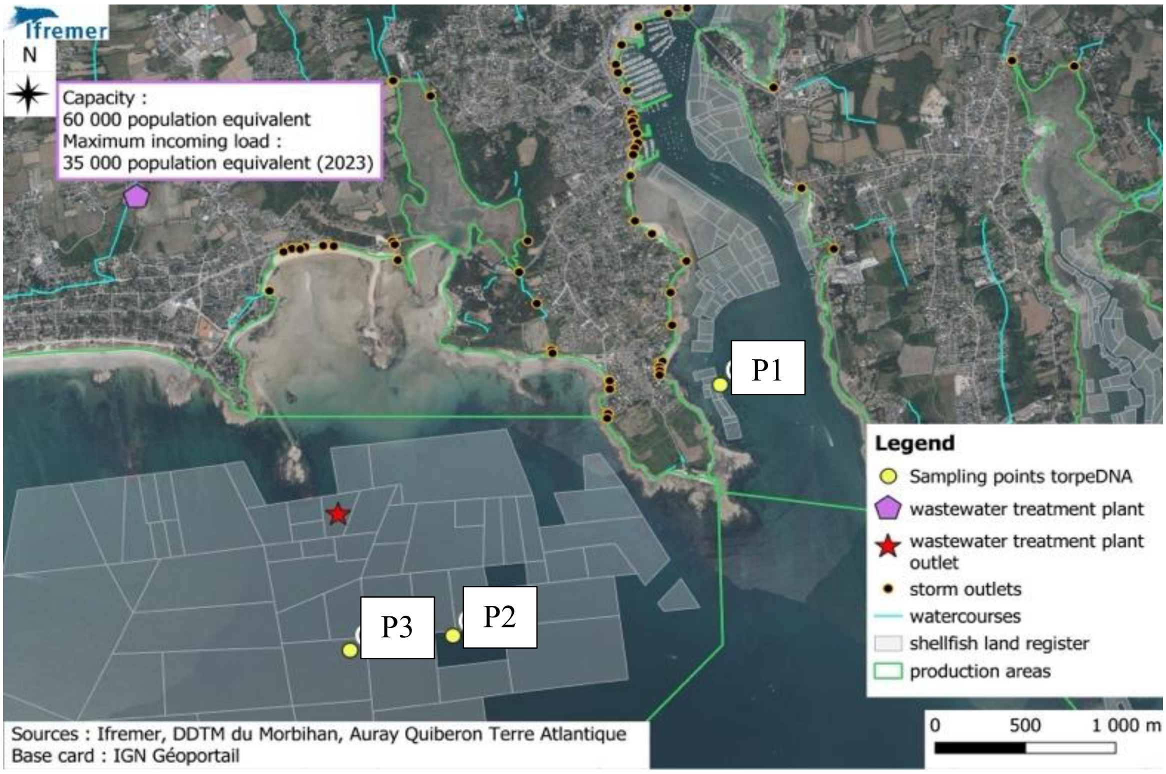

Figure S1A Location of sampling sites (P1-P3) in Quiberon Bay for Case Study 2 (TorpeDNA vs Serial filtration).

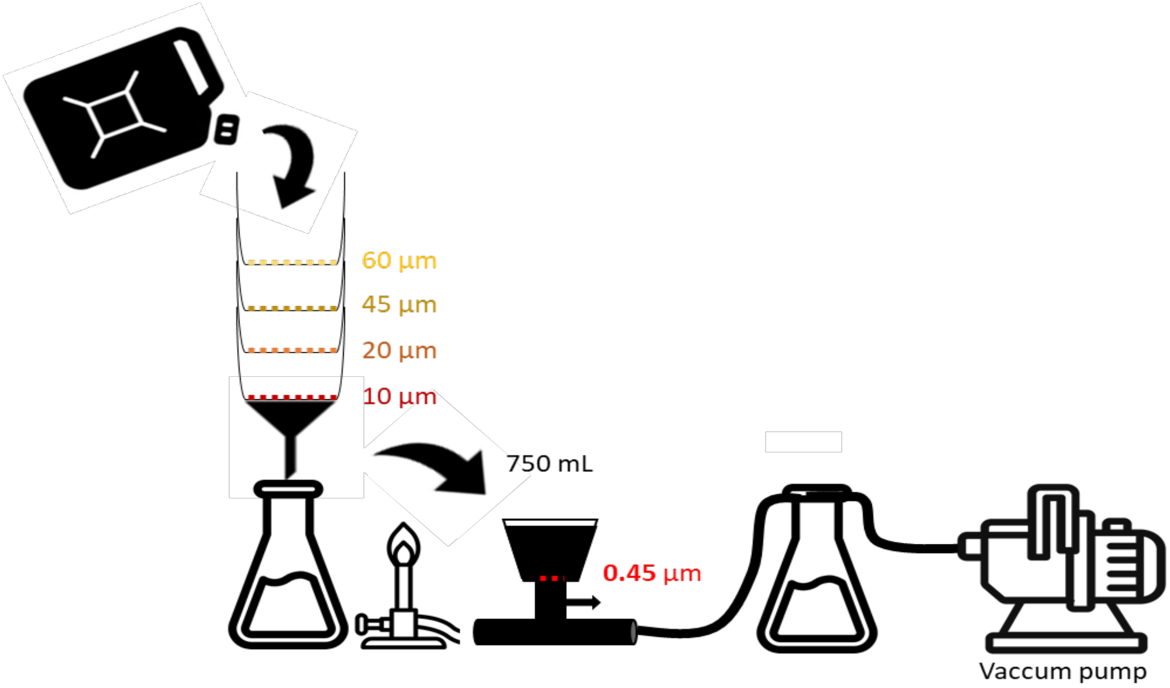

Figure S1B Serial filtration of seawater from 60 µm to 0.45 µm applied in case study 2 under sterile conditions. Only 0.45 µm filters were analyzed for the comparison
