# Supplementary material for: TorpeDNA: a fit-for-purpose eDNA sampling device for marine biodiversity monitoring across applications and scales
Source: PeerJ. 2026 Jun 22;14:e21390. doi: 10.7717/peerj.21390 (PMC13296811; doi:10.7717/peerj.21390)
Supplement: Supplemental Information 2 — Heat map illustrating the detections of different vertebrates (including terrestrial), assigned with the mt16S MarVer3 metabarcode assay, between (A) the TorpeDNA and Waterra sampling methods (case study 1), and (B) the vertebrate taxa detected across the latitudinal transect of case study 3. For each taxa, red indicates positive detection and blue indicates no detection. [file peerj-14-21390-s002.pdf]

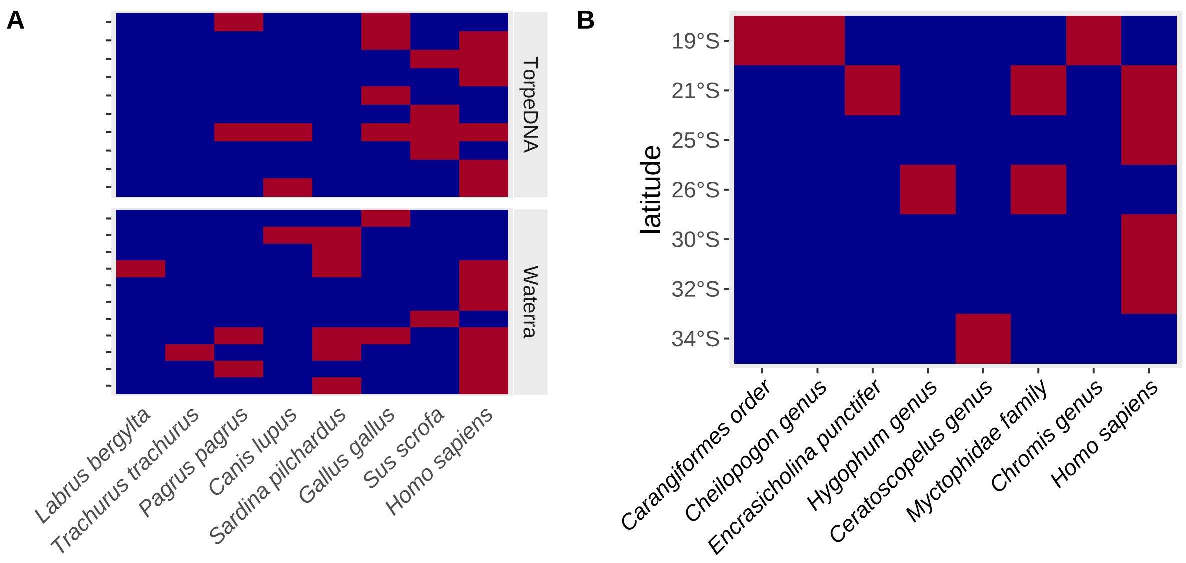

Figure S2 Heat map illustrating the detections of different vertebrates (including terrestrial), assigned with the mt16S MarVer3 metabarcode assay, between (**A**) the TorpeDNA and Waterra sampling methods of case study 1, and (**B**) the vertebrate taxa detected across the latitudinal transect of case study 3. For each taxa, red indicates positive detection and blue indicates no detection.
