# Supplementary material for: TorpeDNA: a fit-for-purpose eDNA sampling device for marine biodiversity monitoring across applications and scales
Source: PeerJ. 2026 Jun 22;14:e21390. doi: 10.7717/peerj.21390 (PMC13296811; doi:10.7717/peerj.21390)
Supplement: Supplemental Information 3 — Relative abundance of the 7 most abundant phyla for the bacterial 16S rRNA (A), eukaryotic 18S rRNA (B) and metazoan COI (C) metabarcodes. The remaining phyla were grouped as “others’. (D, E, F) show the results of Principal Component Analysis for each metabarcode performed on the centred log-ratio-transformed amplicon sequence variant (ASV) abundance data, overlayed with colours representing the latitudinal gradient from New Zealand (−34°) to Fiji (−19°). [file peerj-14-21390-s003.pdf]

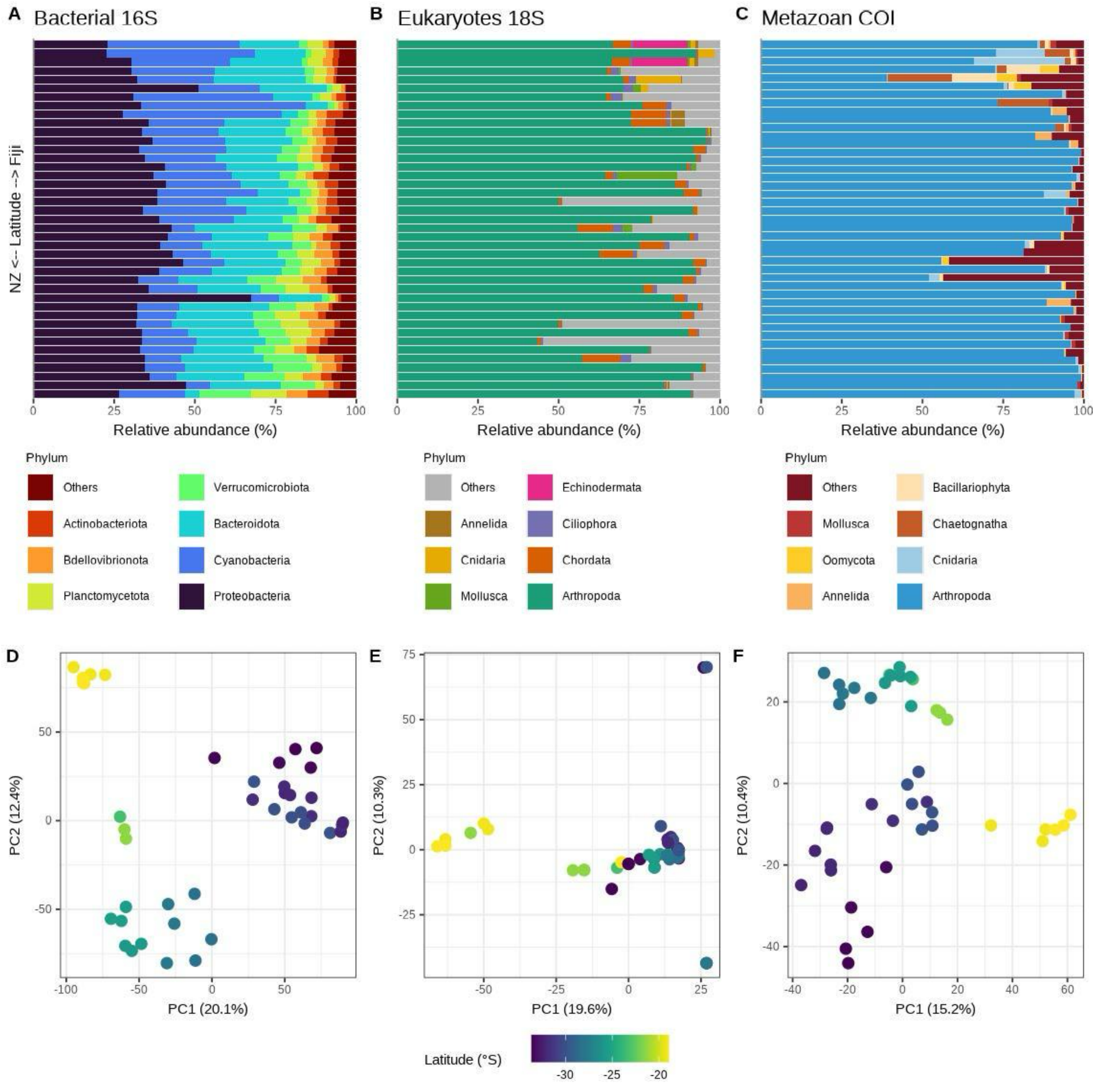

Figure S3. Relative abundance of the 7 most abundant phyla for the bacterial 16S rRNA (**A**), eukaryotic 18S rRNA (**B**) and metazoan COI (**C**) metabarcodes. The remaining phyla were grouped as "others'. **D, E, F**) show the results of Principal Component Analysis for each metabarcode performed on the centred log-ratio-transformed amplicon sequence variant (ASV) abundance data, overlayed with colours representing the latitudinal gradient from New Zealand (-34°) to Fiji (-19°).
