# Supplementary material for: TorpeDNA: a fit-for-purpose eDNA sampling device for marine biodiversity monitoring across applications and scales
Source: PeerJ. 2026 Jun 22;14:e21390. doi: 10.7717/peerj.21390 (PMC13296811; doi:10.7717/peerj.21390)
Supplement: Supplemental Information 4 — Distribution of the number of ASVs across different latitudes for the 16S rRNA (A), 18S rRNA (B) and COI (C) markers depicted in case study 3. [file peerj-14-21390-s004.pdf]

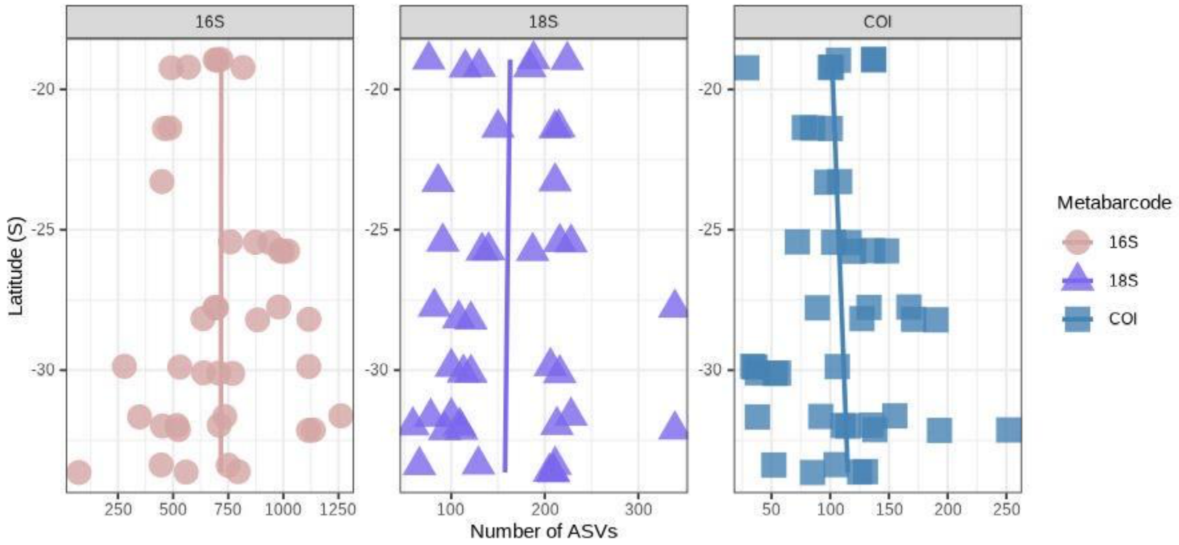

Figure S4. Distribution of the number of Amplicon Sequence Variants (ASVs) across different latitudes for the 16S rRNA (**A**), 18S rRNA (**B**) and COI (**C**) markers depicted in case study 3.
