# Supplementary material for: TorpeDNA: a fit-for-purpose eDNA sampling device for marine biodiversity monitoring across applications and scales
Source: PeerJ. 2026 Jun 22;14:e21390. doi: 10.7717/peerj.21390 (PMC13296811; doi:10.7717/peerj.21390)
Supplement: Supplemental Information 5 [file peerj-14-21390-s005.pdf]

Table S1 List of parts and material components of the TorpeDNA device.

No.	Component	Materials	Specs	Number
1	Nose cone	PLA	3D printed Custom part	1
2	Tube section	UPVC pipe	OD 68.7mm, 337mm long	1
3	Top mount	PLA	3D printed Custom part	1
4	Wing attachment	Stainless Steel 316	Custom made	1
5	Wing	Stainless Steel 316	Custom made	1
6	Wing attachment to tube sections screws	Stainless Steel 316	M5 x 12 316 Button Socket Screw	2
7	Wing to wing attachment screws	Stainless Steel 316	M5 x 12 316 Button Socket Screw	2
8	Fin	PLA	3D printed Custom part	1
9	Fin to tube section screws	Stainless Steel 316	M5 x 12 316 Button Socket Screw	2
10	Threaded insert to tube section	PLA	3D printed Custom part	1
11	End cap	PLA	3D printed Custom part	1
12	O ring	Nitrile	BS228 or AS568-218	1
13	Mesh screen insert	Stainless Steel 304	Custom made	1
