# Supplementary material for: TorpeDNA: a fit-for-purpose eDNA sampling device for marine biodiversity monitoring across applications and scales
Source: PeerJ. 2026 Jun 22;14:e21390. doi: 10.7717/peerj.21390 (PMC13296811; doi:10.7717/peerj.21390)

Supplementary Material 1 Improved TorpeDNA features obtained from Blender Ltd (Auckland, NZ), following computational fluid dynamics (CFD) analyses in February 2024, which reduced the drag of the device by 55%.

Proposed TORPeDNA Prototype Changes.

DATE: 07 February 2024

Inspected/Tested By: Gordon Robinson

Product: CAW002-1000 TORPeDNA

Summary

A CFD analysis of the original prototype CAD model was carried out to find areas to improve the drag performance of the prototype unit. All the below proposed changes were made without fundamentally changing the design already proven in the last prototypes, but rather focusing on improving flow through the unit, and reducing drag at various angles of attack

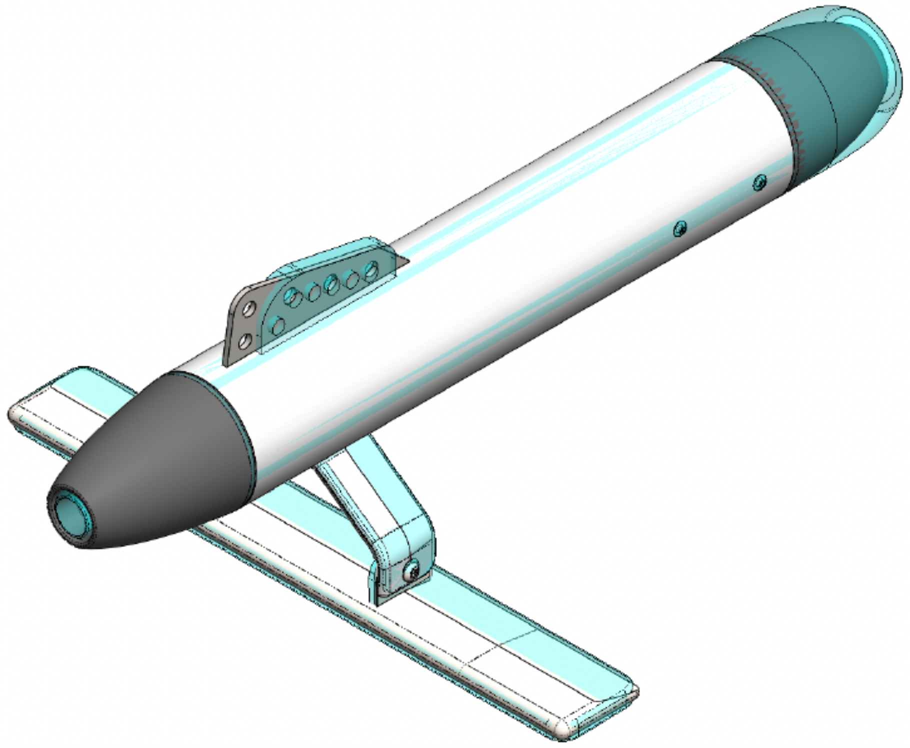

Supplement: Supplemental Information 7 — Improved TorpeDNA features obtained from Blender Ltd (Auckland, NZ), following computational fluid dynamics (CFD) analyses in February 2024, which reduced the drag of the device by 55%. [file peerj-14-21390-s007.pdf]
