# Supplementary material for: TorpeDNA: a fit-for-purpose eDNA sampling device for marine biodiversity monitoring across applications and scales
Source: PeerJ. 2026 Jun 22;14:e21390. doi: 10.7717/peerj.21390 (PMC13296811; doi:10.7717/peerj.21390)
Supplement: Supplemental Information 8 — Step-by-step protocol used by Citizen Scientists as part of the Citizens of the Sea initative for the collection and isolation of eDNA samples using the TorpeDNA device. [file peerj-14-21390-s008.pdf]

torp*eDNA*
eDNA Collection Protocol

SAFETY FIRST

Ensure that participating crew members are wearing PFDs and securely clipped in before deploying and while using the device.

Step 1

Prepare for sampling collection

Before starting daily sampling tows:

- Ensure the sea conditions are favourable (<2m swell recommended) and good visibility.
- Choose a sampling time when maintaining a boat speed at <12 knots is feasible. (ideal speed = 5 knots, ideal sampling time = 10am - 2pm).
- Crew collecting samples should feel well and be protected from sun, wind, and cold.

Setup dry lab area and prepare the torp*eDNA*:

Gather materials (gloves, tweezers, anti-bacterial wipes, filter box, KimWipes). Lay out two KimWipes on a flat, clean surface.

1. Put lab gloves on and keep on throughout all steps of this protocol.
2. Ensure the torp*eDNA* device is securely fastened to the line, line to the vessel, and a knife is close to hand.
3. Rinse torp*eDNA* by towing for 5 seconds.

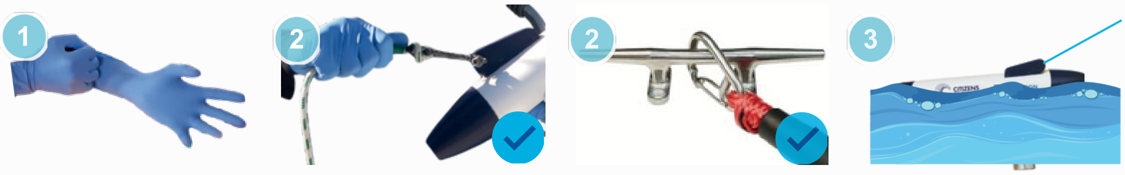

Step 2

Insert Nylon Filter

1. Unscrew torp*eDNA* end-cap and place it on KimWipes in the dry lab area.
2. Clean both tweezers using anti-bacterial wipes.
3. Use tweezers to remove the black O-ring.
4. Open one tube from the filter box and use tweezers to grab the filter.
5. Place filter inside of end-cap – make sure it is flat and aligned with the grid.
6. Lock the filter in place with black O-ring (minimize touching the filter).
7. Carefully screw the end-cap onto the torp*eDNA*.

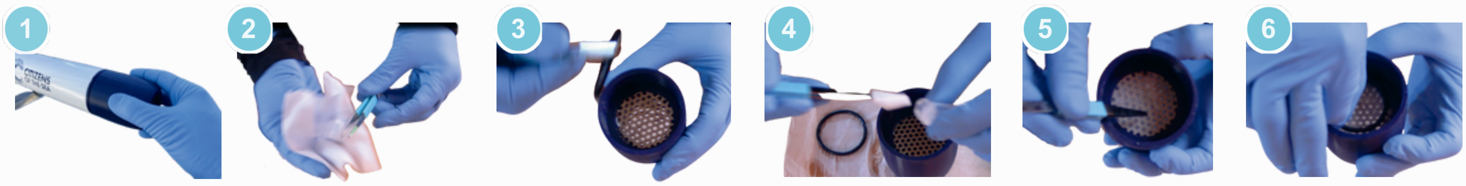

Step 3

Collect eDNA

1. Plan to launch torpe*DNA* device when <12 knots boat speed can be maintained (ideal collection speed is 5 knots).
2. Double-check the torpe*DNA* device is securely fastened to the line, and line to the vessel
3. Confirm with Skipper/Helm, clear to launch (no vessels or objects visible ahead)
4. Deploy the torpe*DNA*, feed out 15m line, and tow for exactly 5 minutes.
5. Recover the device and hold it upright until all water drips through the end-cap.

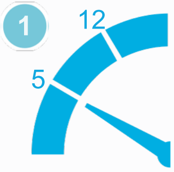

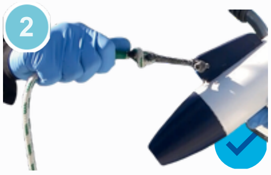

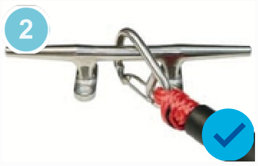

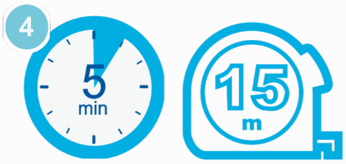

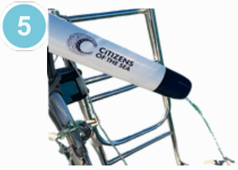

Step 4

Store Filter

1. Unscrew the end-cap and place it on KimWipes in the dry lab area.
2. Remove the sample box from the fridge and take out an unused sample tube.
3. Log the sample using the Survey123 app **(see attached logging guide).**
4. Using the tweezers, remove the O-ring from end-cap and place on KimWipes.
5. Using tweezers, fold over the filter twice and transfer it to the scanned sample tube.

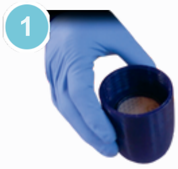

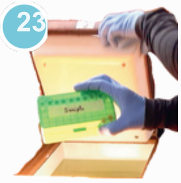

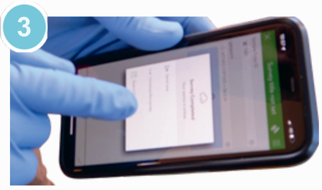

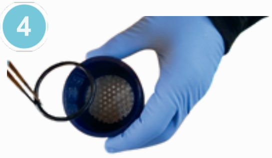

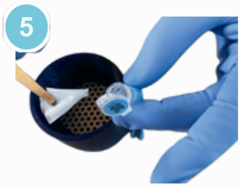

Step 5

Repeat

Repeat Steps 2, 3 and 4.

After the last sample of the day, place the sample box back into your fridge. Safely store gloves, tweezers, anti-bacterial wipes, KimWipes, and filter box until the next day’s sampling.

Log Daily Observations using the Survey123 app **(see attached logging guide).**

Important Note: On the last day of your consecutive eDNA sampling, transfer one individual filter (from the filter box) into each of the three control sample tubes with orange QR codes. These also need to be entered in the Survey123 app.

torpe*DNA* is a registered trademark owned by the Cawthron Institute. All rights reserved
