# Supplementary material for: TorpeDNA: a fit-for-purpose eDNA sampling device for marine biodiversity monitoring across applications and scales
Source: PeerJ. 2026 Jun 22;14:e21390. doi: 10.7717/peerj.21390 (PMC13296811; doi:10.7717/peerj.21390)
Supplement: Supplemental Information 9 — Parameters for taxonomic assignments (blastn_taxo_assignment function of the biohelper R package) used in this study for the 18S rRNA (18S), Cytochrome C Oxidase I (COI), and mitochondrial 16S (MarVer3) markers. [file peerj-14-21390-s009.pdf]

Supplementary Material 3 Parameters for taxonomic assignments (blastn_taxo_assignment function of the biohelper R package) used in this study for the 18S rRNA (18S), Cytochrome C Oxidase I (COI), and mitochondrial 16S (MarVer3) markers.
follow:

18S: minSim = 100,
minCov = 80,
pident = "after",
pgenus = 97,
pfamily = 95,
porder = 87,
pclass = 83,
pphylum = 81,
pkingdom = 79

COI & **MarVer3**: minSim = 97,
minCov = 80,
pident = "after",
pgenus = 95,
pfamily = 87,
porder = 83,
pclass = 81,
pphylum = 79,
pkingdom = 71
